# Decision making in Parkinson's disease: An analysis of the studies using the Iowa Gambling Task

**DOI:** 10.1111/ejn.15497

**Published:** 2021-11-09

**Authors:** Laura Colautti, Paola Iannello, Maria Caterina Silveri, Alessandro Antonietti

**Affiliations:** ^1^ Department of Psychology Catholic University of the Sacred Heart Milan Italy

**Keywords:** decision making, impulsivity, Iowa Gambling Task, Parkinson's Disease, reward

## Abstract

In Parkinson's disease (PD) impairments in decision making can occur, in particular because of the tendency toward risky and rewarding options. The Iowa Gambling Task has been widely used to investigate decision processes involving these options. The task assesses the ability to manage risk and to learn from feedback. The present paper aims at critically examining those studies in which this task has been administered to PD patients, in order to understand possible anomalies in patients' decision processes and which variables are responsible for that. A meta‐analysis has been conducted as well. Features of the task, sociodemographic and clinical aspects (including daily drugs intake), cognitive conditions and emotional disorders of the patients have been taken into account. Neural correlates of decision‐making competences were considered. It emerged that PD patients show a trend of preference toward risky choices, probably due to an impairment in anticipating the unrewarding consequences or to an insensitiveness to punishment. The possible role played by dopamine medications in decision making under uncertain conditions, affecting basal ganglia and structures involved in the limbic loop, was discussed. Attention has been focused on some aspects that need to be investigated in further research, in order to delve into this issue and promote patients' quality of life.

## Graphical abstract

A meticulous analysis of the studies published from 2000 about decision making in Parkinsonian patients has been led. Attention was paid both to the variables influencing decision making in Parkinsonian patients and to the neurobiological correlates of decision making. An explanation of Parkinsonian patients’ behavior in the Iowa Gambling Task and suggestions for further studies have been proposed.

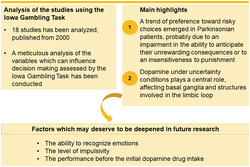

**Abbreviations**
DBS: deep brain stimulation
dlPFC: dorsolateral prefrontal cortex
ERPs: event‐related potentials
fMRI: functional magnetic resonance imaging
ICDs: impulse control disorders
IGT: Iowa Gambling Task
MDRS: Mattis Dementia Rating Scale
MMSE: Mini‐Mental State Examination
OFC: orbitofrontal cortex
PD: Parkinson's disease
PET: positron emission tomography
PFC: prefrontal cortex
QUIP: Questionnaire for Impulsive‐Compulsive Disorders in Parkinson's disease
SPN: stimulus preceding negativity
vmPFC: ventromedial prefrontal cortex

## 1 INTRODUCTION

### 1.1 Decision making in PD

Decision making is a fundamental activity in humans' life because it occurs in a variety of situations and has a crucial role in solving adaptively everyday problems and dealing with interpersonal, social and moral issues and dilemmas. Therefore, impairments or alterations in the normal way of making decisions have heavy consequences in managing practical and existential matters. Overt and dramatic modifications in the decision‐making process appear in patients with Parkinson's disease (PD), mostly as a consequence of dopamine replacement therapy and stimulation of D3 receptor (Heiden et al., 2017; Ibarretxe‐Bilbao et al., 2009; Voon, 2017). The most common consequence is pathological gambling, namely, the persistent, recurrent and impulsive tendency to gamble despite the severe repercussions that repetitive high losses of money have on personal, familiar and professional life (e.g., Dodd et al., 2005; Drapier et al., 2006; Driver‐Dunckley et al., 2003; Gschwandtner et al., 2001; Molina et al., 2000; Seedat et al., 2000; for a review, Biars et al., 2019; Gallagher et al., 2007). Such aberrant behaviours, which affect 2.3% to 9.3% of PD patients (Biars et al., 2019; Vitale et al., 2011), have been mainly associated to dopamine tone and dopamine dysregulation, although such disorders do not affect all the patients who take the abovementioned medications (Kobayakawa et al., 2017), suggesting the presence of other factors that may contribute to their development (Kobayakawa et al., 2017; Valença et al., 2013). More generally, PD patients display mild to moderate, non‐pathological increasing drive toward rewards (Cools, 2006).

It was conjectured that the tendency toward risk taking and the inability to learn from negative feedback—which are so blatant in pathological gambling—are common characteristics of PD patients so as to affect their decision processes, which involve different anatomic substrates—such as the striatum, the amygdala, the orbitofrontal cortex (OFC), both lateral and medial portions, the anterior cingulate cortex and, more generally, the prefrontal cortex (PFC) (Clark et al., 2004; Gleichgerrcht et al., 2010; Ibarretxe‐Bilbao et al., 2009; Salvatore et al., 2021)—and require their integration (Bechara, 2005; Clark et al., 2008; Zeeb & Winstanley, 2011).

### 1.2 The IGT

The Iowa Gambling Task (IGT) (Bechara et al., 1994) is the mostly employed task to test experimentally decision‐making processes in pathological conditions (Bechara et al., 2005) and in PD specifically. This task is so used because it is slightly correlated with the results of other executive function tasks, it is considered a measure about the functioning of PFC, hardly ever healthy subjects fail to perform it and it is possible to account punishment–reward conditions (Balconi et al., 2014; Poletti, Cavedini, & Bonuccelli, 2011).

In the IGT, the participant is presented with four decks of cards. Each card reports, on the side hidden to the participant, the amount of money that is won and, if it is the case, the amount of money that is lost as a consequence of having selected that card (for instance, ‘Win $100 and lose $1,250’). The participant, who is given an initial sum of money, is required to increase that sum by selecting cards from the four decks. The participant is usually asked to make 100 choices. The four decks are designed so that, over the course of several choices, two of the decks are advantageous (or safe) because they produce a net win, whereas the other two decks are disadvantageous (or risky) because they produce a net loss. In fact, two decks allow the participant to win, and also to lose, small sums of money, so producing, after many selections, a positive outcome (if the player always chooses these decks, his/her total gain will be larger than the total loss), whereas the other two decks are disadvantageous because they give players high winnings and imply very high losses, so that, across several selections, the total loss is larger than the total gain. The two advantageous decks differ from each other in the frequency of losses, and the same is true for the disadvantageous decks.

When engaged in the task, players usually fail to identify the advantageous decks in the first choices, by showing selections near the chance level. However, as the game goes on, they generally choose more and more frequently cards from the advantageous decks.

In order to record such a change in behaviour, experimenters often analyse separately four to five blocks of consecutive 25–20 trials. Many authors instead preferred analysing only the latter blocks of the task, dividing the IGT into two measures: decision under ambiguity (namely, in the former blocks, in which the participant does not know much about reward/punishment ratio of each deck) and decision under risk (in the latter blocks, when the advantageous/disadvantageous features of the decks become clear to the participant) (Brand, Recknor, et al., 2007; Buelow et al., 2014).

It is worth noting that a preference for safe decks emerges before the players become aware of the differences existing among the two kinds of decks. In fact, players are not able to verbalize any possible reason supporting their selections even though they choose more often cards from the advantageous decks. The occurrence of a sort of tacit, unconscious form of identification of the opposite features of the two kinds of decks, is testified by skin conductance responses, which indicate an increased psychophysiological emotional anticipatory response when the participant selects a card from a disadvantageous deck (which involves the opportunity to gain more, but also the risk of losing much more), so showing that the nervous system ‘recognizes’ the risky nature of that deck (Bechara et al., 1997). Decision‐making competences, mental flexibility, impulse control, reversal learning and reward/punishment sensitivity are necessary to successfully achieve the task (Fellows & Farah, 2005; Mimura et al., 2006; Salvatore et al., 2021).

### 1.3 Aims

The aim of this work was to examine experimental studies involving PD patients in which the IGT is administered. More precisely, the first goal was to check whether anomalies in the IGT emerge in PD patients in the absence of other pathologies. The second one was to identify which variables can be responsible for such anomalies. The third one was to understand the possible mechanism(s) which support the anomalies in question.

The paper has been organized as follows. After having declared the procedure and criteria applied to identify and select the studies to be taken into account, all studies in which the IGT was presented to PD patients have been reviewed by following a chronological order and their main variables have been summarized in a series of tables. A meta‐analysis of the considered studies was carried out, to verify the extent to which PD patients show a different trend in choices in the IGT compared with healthy controls. Afterwards, the performance in the IGT was critically analysed according to the parameters of the task recorded by the authors, the features of the task and the patients' characteristics (namely, the demographic variables, the medical treatment and the cognitive and emotional status). Correlations between scores in the IGT and other measures recorded in the studies were also taken into account. After having analysed thoroughly the empirical findings, a possible explanation of the peculiar behaviour of PD patients in the IGT is proposed, by discarding alternative interpretations which are not supported by evidence. Such an explanation is expanded by considering data coming from the neurobiological counterparts of decision making in PD patients (as compared with healthy individuals). Finally, some conclusions are drawn.

## 2 SELECTION OF THE STUDIES

In this paper, only studies in which PD patients have been matched to comparable healthy controls have been reviewed (in only one case, two groups of idiopathic PD patients, with or without alexithymia, were compared with each other; Poletti, Frosini, et al., 2011).

Studies published from 2000 have been collected through PubMed and PsycINFO entering the keywords: Iowa Gambling Task AND (Parkinson's Disease) AND (decision making OR decisional processes). Moreover, a cross‐analysis of the references of the articles has been made. Two categories of studies have been excluded: studies where idiopathic PD patients with impulse control disorders (ICDs) or pathological gambling have been compared with idiopathic PD patients without such behavioural outcomes and studies in which idiopathic PD patients have been matched to comparable idiopathic PD who received deep brain stimulation (DBS), before and after the surgical intervention. A total of 18 studies were analysed.

Table 1 reports a brief description of the considered studies. The performances of the clinical groups in the IGT, as compared with those of the control groups, have been summarized in Table 2. Table 3 reports an analysis of the features of the versions of the IGT which was used in the studies. Table 4 shows the main characteristics of the clinical groups investigated. Tables 5a and 5b describe in detail the relationships between the performances in the IGT and demographic, clinical and pharmacological variables, as well as the overall and fine‐grained measures of cognitive functioning and of mood/emotional state of the patients. In the considered studies, dementia has been always excluded on the basis of a psychiatric assessment and/or of the administration of specific screening tests for dementia and/or for general intellectual level.

**TABLE 1:** Brief description of the studies

Study	Aim	Inclusion criteria of PD patients	Results
Stout et al. (2001)	Investigate decision making in HD, PD and HC.	Lack of severe dementia; an eighth grade education or higher; absence of drug or alcohol abuse; absence of other neurological diagnoses than HD or PD; absence of major psychiatric diagnoses.	HD patients showed poor performance in the IGT (by failing to select preferably advantageous decks in the second part of the task), PD patients performed as well as the HC participants, by learning to choose cards from the safe decks as the task proceeded, and by showing—as controls—a risk‐aversion attitude in the last phases of the game. The scores of the cognitive functioning were not associated with the number of advantageous selections in the IGT. Performance in the IGT failed to correlate with apathy, disinhibition and executive dysfunctions. The patterns of findings were the same by excluding patients who received neuroleptics, so proving that pharmacological treatment has no relation to the performance in the IGT.
Czernecki et al. (2002)	Investigate behavioural responses in PD patients, comparing with HC, and evaluate the influence of dopaminergic therapy.	Good reactivity to l‐Dopa; lack of dementia or depression; ability to be tested both in the ‘on’ state and in the ‘off’ state (after about 12 h of therapeutic withdrawal).	PD participants were engaged in the IGT twice: once in the ‘on’ state and the other time in the ‘off’ state. As far as the choice of advantageous decks was concerned, in the first round, no differences between PD patients and HC emerged: Both subsamples moved progressively from the preferential selection of risky decks to that of safe decks. By contrast, the second time participants played the IGT, HC improved further their performance whereas PD patients chose advantageous decks in the second part of the game to a similar extent as they did in the first round, with no further improvement. Starting from the third block of 20 trials of the first round and along all the blocks of the second round, PD patients chose a higher number of advantageous than disadvantageous decks, showing to have early developed risk aversion. Authors suggested that PD patients fail to progress in the IGT due to a drop in motivation, attention, sensitivity to reinforcement or inability to reach, as occurred in HC, an explicit awareness of the features of the two kinds of decks. Age and education had a little effect on the global IGT performance, which was negatively correlated to scores in the frontal function test. The age at the onset of the disease, its duration and motor skills failed to be related to scores in the IGT. The ‘on’ versus ‘off’ state failed to influence performance, showing no direct effects of dopamine treatment on the decision‐making task.
Thiel et al. (2003)	Investigate a small sample of PD patients performing the IGT while data from PET were recorded.	Lack of dementia; absence of other neurological diagnoses; absence of psychiatric diagnoses.	No significant difference between PD patients and HC was found in the number of cards selected from advantageous and disadvantageous decks in the IGT, even though the former ones tended to choose more risky decks than the later ones (in any case, both groups chose a higher overall number of risky than safe cards). PET data showed a consistent bilateral activation of the dlPFC, the right mesial OFC, the right cingulate and the left caudate in HC during the IGT. In PD patients, a bilateral activation regarding the dlPFC and the left caudate was remarkable as well, while the OFC, especially in the mesial portion, was ipoactivated and the right thalamus was deactivated, suggesting an impairment of this cortical–subcortical loop, specifically in the basal ganglia loop, which links the abovementioned regions to the thalamus through the ventral striatum. In other words, it seemed that in PD patients, the limbic loop was impaired, whereas the cognitive loop was more preserved.
Perretta et al. (2005)	Investigate the performance of PD patients in the earlier or later stages of the disease.	Lack of dementia or learning disabilities; absence of other neurological diagnoses; absence of psychiatric diagnoses.	The PD groups behaved similarly to HC in the first part of the IGT, but in Blocks 5 to 7 (out of 10), they failed to select the advantageous decks to a large extent as the HC did (differences between the clinical and control groups were statistically significant only in Block 7). An overall inspection of the rates of selection of safe versus risky cards made by the PD subsamples highlighted that also these groups of participants learned to prefer advantageous decks and missed the tendency to choose risky cards in the last blocks, so behaving in a way that was similar to the controls' one. In the early PD group, depression was correlated significantly with the IGT. The cognitive test showed no correlation with the IGT, nor other significant correlations emerged.
Mimura et al. (2006)	Investigate decision making in PD patients and possible links with cognitive abilities.	Lack of dementia; absence of alcohol abuse; absence of other neurological diagnoses; absence of psychiatric diagnoses.	At the end of the IGT, PD patients obtained a lower amount of money as compared with HC, so showing to be lacking in risk evaluation skills. Regarding the number of times participants chose advantageous decks, in the first part of the game, no difference emerged between the two groups. In the second part, HC chose advantageous decks more frequently than PD patients, even though the difference between the two groups was only approaching statistical significance (*p* = .08) and the difference between the number of times PD patients selected safe cards and those they selected risky cards was quite null. Performance in the IGT resulted to be positively correlated only with the mindreading test, in which PD patients identified a significantly lower number of mental states than HC. No other significant correlations emerged, neither with executive functioning (in which PD performed significantly worse than HC). In the authors' opinion, impairments in the IGT cannot be attributed to executive function deficits, as well as to lower levels of intelligence or depression, but to weaknesses in mindreading.
Pagonabarraga et al. (2007)	Delve into the relationship between limbic and cognitive dysfunction in PD patients, as assessing decision making and cognitive abilities. Patients were divided into two subsamples according to their response—stable versus fluctuating—to oral intake of levodopa.	Lack of dementia; absence of mood or psychiatric disorders; absence of abnormalities on noncompensated systemic diseases, neuroimaging studies or blood testings.	The mean total score in the IGT was lower in PD patients (who overall chose a higher number of disadvantageous than advantageous cards) as compared with HC (who chose a higher number of advantageous than disadvantageous cards). Moreover, whereas HC showed an increasing preference toward advantageous decks along the trials, PD patients always selected preferentially disadvantageous decks. No differences in the IGT between stable and fluctuating PD patients emerged. Moreover, statistically significant negative correlations between the IGT and general intellectual functioning, delayed free‐recall visuospatial abilities and verbal fluency were found. No other significant correlation between measures emerged in PD patients. The authors suggested that the negative correlation between the IGT performance and general intellectual efficiency and working memory can be explained by hypothesizing that the better the cognitive status is, the more prone the individual is to take risk. No differences in the IGT between stable and fluctuating PD patients emerged. This finding, according to the authors, disconfirm the claim that PD patients' decisional process in the IGT is due to a mechanism that is similar to that implicated in the development of ICDs, namely, the raising in sensitivity of the limbic circuits due to the postsynaptic changes for discontinuous stimulation of dopaminergic drugs, nor is due to a direct effect of dopamine, also because authors found no significant correlation between the IGT performance and the dosage or the type of drugs. These assumptions about the medication seem to be consistent with the results of Czernecki et al. (2002), who found no differences in the IGT performance between patients in ‘on’ and ‘off’ states.
Kobayakawa et al. (2008)	Investigate decision making in PD patients, compared with HC, recording skin conductance responses.	Lack of dementia; absence of an ongoing or past history of impulse control disorder.	In the IGT, both the total score and the total amount of money left at the end of the game were lower in PD patients as compared with HC. As far as the choice of the two kinds of decks was concerned, while in the first block of 20 trials no difference between PD patients and controls emerged, starting from the second to the fifth blocks HC chose more frequently the advantageous decks, whereas the PD patients showed a slightly increasing preference for risky decks. Cognitive and executive function tests failed to be correlated to performance in the IGT. Only a positive statistically significant correlation between the IGT and depression was found: The more the patients were depressed, the more they selected advantageous decks. The skin conductance response of 13 patients and 12 controls during the IGT was recorded. Patients showed lower skin responses after the choice of the deck and before knowing the outcome of the choice than controls; the same was true by analysing skin conductance responses recorded after the participant had known if the card selected produced a gain or a loss. HC, but not PD patients, exhibited higher skin responses in correspondence of the choice of risky decks as compared with the choice of safe decks. In other words, PD patients experienced less emotional responses and were less sensitive to gains and losses.
Euteneuer et al. (2009)	Investigate decision making in PD patients, exploring electrodermal activity.	Lack of dementia; absence of other neurological diagnoses; absence of major depression disorders or psychiatric diagnoses.	Regarding IGT, a lower outcome and a lower use of negative feedback were found in the PD group, but the difference in performance with HC was not significant. Moreover, authors found no correlations between the performance in the IGT and the scores in neuropsychological tests (including executive functions), nor the features of the illness nor the medication doses. Regarding electrodermal response, in the HC, anticipatory responses were higher before choosing disadvantageous decks than advantageous decks in the IGT whereas in idiopathic PD patients this pattern failed to emerge. Responses after losses but not after gains were significantly reduced in PD patients, who showed an impairment of the sensitivity for negative feedback, but not for positive feedback.
Kobayakawa et al. (2010)	Investigate the possible causes of impairments in decision making in PD patients, focusing on the sensitivity to reward and punishment.	Absence of impulsive control disorders or psychiatric diagnoses.	Two versions of the IGT were administered. The first one was consistent with the traditional one; after 3–6 months, a modified version was proposed, in which advantageous decks led to immediate large losses and delayed larger wins and disadvantageous decks led to immediate small losses and delayed smaller wins (in other words, for the advantageous decks the immediate large losses did not hamper net gains in the long term). The two versions had been devised to highlight whether difficulties shown by PD patients in the IGT are simply due to a hypersensitivity to immediate decisional outcomes (if an evident impaired performance was present in both versions of the task) or difficulties in balancing reward and punishments are also involved (if differences in the performance between the two versions emerged). Results showed that, in the traditional version of the IGT, patients scored significantly lower from the third block with a lower total gain, and the choice pattern was more disadvantageous if compared with HC. Instead, in the modified version, there were no significant differences neither in the choice pattern nor in the total gain between the two groups. Authors included among the limits of their study the methodological choice to administer the modified version of the IGT after the original one for all the patients, so it cannot be excluded that the second performance had been influenced by the previous one, although 3 to 6 months elapsed between the two versions of the task. Anyway, they stated that decision‐making impairments may be bound to an impairment in the ability to balance rewards and punishments and to hypersensitivity to reward and/or lack of sensitivity to punishment.
Poletti et al. (2010)	Investigate decision making in de novo PD patients, compared with HC.	Lack of dementia; absence of abnormalities on noncompensated systemic diseases, or neuroimaging studies.	Both de novo PD patients and HC chose cards from advantageous decks from the third block of five, without significant differences in the performance. De novo patients performed similarly to the control group, recording a positive mean score in the IGT. Authors concluded that the dopaminergic therapy can affect learning from negative feedback. An interesting correlation emerged in the PD group: The higher was the score on the self‐control subscale of impulsivity, the lower was the IGT total score, highlighting the importance of impulsiveness in the task.
Poletti, Frosini, et al. (2011)	Investigate the responses to the IGT in de novo PD patients, accounting the degree of alexithymia and of depression.	Lack of dementia; absence of abnormalities on noncompensated systemic diseases or neuroimaging studies.	No significant differences in the IGT total score occurred in the two subgroups of de novo patients (even though the alexithymic group performed better than the other one), but in the third block of cards, the alexithymic group outperformed significantly the other one. No differences between groups emerged comparing patients with mild depression and patients without it, probably because no patients with moderate or severe depression were included in the sample. Moreover, the alexithymia subscale (assessing difficulty in identifying feelings) correlated positively with the performance in the IGT in the second block of cards and negatively with the performance in the fourth and the fifth blocks. Authors claimed that a difficulty in identifying emotions related to wins/losses can bring to the adoption of a conservative strategy and hypothesized that alexithymia could modulate decision‐making processes in the IGT.
Gescheidt et al. (2012)	Investigate decision‐making processes in PD patients with early onset (≤45 years old), compared with matched HC.	Lack of dementia or impairments in executive functions; absence of severe depression; absence of a history of pathological gambling.	Data showed a slight difference in the IGT performance between groups, with PD patients obtaining lower scores. The authors noted that in the first part of the task, the choices were random, but from the second to the fifth part of the task, the HC tended to choose cards from more advantageous decks compared with the clinical one. It is worth noting that the two groups adopted different strategies to complete the task. While the HC regularly selected the two advantageous decks, even when monetary penalty occurred, the PD patients tended to change preferences more often, frequently choosing the disadvantageous decks. Although both groups preferred decks with lower penalty frequencies, PD patients selected the decks with the higher winning values, whereas the HC tended to choose the advantageous decks, showing a tendency to use strategies based on long‐term outcomes.
Gescheidt et al. (2013)	Investigate the functional anatomy (through fMRI) of decision‐making process in PD patients, compared with HC.	Lack of cognitive impairment; absence of severe depression; absence of a history of pathological gambling.	Reduced activation of the left putamen was observed in PD patients as a reaction to penalty. In addition, a decreased functional connectivity was observed by psychophysiological interactions analysis between the right globus pallidus and the left anterior cingulate gyrus in the PD group, whereas increased connectivity between the same structures was documented in HC after penalty. The authors concluded for dysfunctional limbic frontostriatal circuitry in PD leading to an insufficient negative reinforcement after a loss.
Mapelli et al. (2014)	Investigate neural correlates of feedback processing in decision making, using ERPs.	Lack of dementia; absence of severe depression; absence of other neurological diagnoses; absence of psychiatric diagnoses; use of psychiatric and neurological medications.	Both PD patients and HC were given the computerized IGT during EEG registration. Continuous EEG data were epoched to analyse ERPs within a time‐window starting before and ending after feedback presentation. The average was performed separately for positive and negative feedback. Results highlighted lower performances in the PD group compared with HC, as well as a preference for disadvantageous decks, a lower learning rate from feedback and a difficulty in following a strategy to perform the task. The performance was neither correlated with any variable bound to the clinical sample's sociodemographic conditions nor to the disease. Moreover, PD patients reported a similar ERPs morphology after the condition of win and loss while in HC, a significant difference between these two conditions occurred. Thus, authors indicated that the worse PD group's performance might be due to an incorrect evaluation of the outcomes, possibly due to an abnormal feedback processing. ERPs were larger on anterior sites in HC at variance than in PD patients indicating, in this population, a posterior shift. This was interpreted by the authors as a different recruitment of neural resources in PD probably due to dysfunction of the frontal cortex.
Buelow et al. (2014)	Investigate the influence of apathy on decisional processes and of dopaminergic therapy in PD patients.	Lack of dementia or mild cognitive impairment; absence of severe depression, absence of significant head injuries; absence of a history of heavy substance abuse or of pathological gambling.	Authors considered only the latter two blocks of the IGT for the analyses. The PD group selected more frequently cards from the disadvantageous deck with higher immediate profits and low frequency in losses, compared with HC. By dividing PD patients according to the variable ‘apathy’, the mentioned behaviour was significantly more frequent in patients with apathy than in patients without apathy and in HC. Data suggested that apathy may be a parameter to take into account when investigating decision processes in PD patients.
Xi et al. (2015)	Investigate decision making and affective Theory of Mind in PD patients, compared with HC.	Lack of dementia; absence of severe depression; absence of other neurological diagnoses.	PD patients underscored the mindreading test as compared with HC. Regarding the IGT, although no significant difference between the two groups emerged in the overall performance, after two blocks of trials, PD patients' net score was below 0, while the HC's one was above 0. The total number of advantageous cards selected in the IGT resulted to be positively correlated only with the mindreading test score, suggesting, together with Mimura et al.'s (2006) findings, that the choices under ambiguous conditions might be linked to the ability to recognize emotional mental states.
Kobayakawa et al. (2017)	Investigate the neural correlates of decision making in PD patients, through a voxel‐based morphometry study.	Lack of dementia; absence of a history of impulse control disorder or other psychiatric disorders.	A reduced grey matter thickness was documented in the medial orbitofrontal cortex, left inferior temporal cortex and right middle frontal gyrus in PD patients compared with HC. By a regression analysis, the authors also found in PD patients a correlation between the lateral orbitofrontal volume and the performance obtained in the IGT, suggesting that this region might be related to the evaluation of punishment, which is a variable supposed to influence decision making in PD patients.
Kjær et al. (2020)	Investigate possible impairments in decision making in PD patients compared with HC and delve into the possible effects of dopaminergic medication on the IGT performance.	Lack of dementia; absence of severe depression; absence of psychiatric or other neurological diagnoses; absence of drug or alcohol abuse; absence of deep brain stimulation implant.	Results showed that the PD group chose more frequently the disadvantageous decks, compared with HC. From regression analyses, it also has been found that the dopamine medication predicted the IGT scores, but only a very small portion of variance was explained (*R* ^2^ = .04, *F* = 68.28, *p* < .001). Authors were the first to report data which cannot exclude a possible (direct or indirect) effect of dopamine. Furthermore, they had counted the number of shifts after wins and losses, finding no significant difference between the two groups. In addition, the researchers had led a more detailed analysis on the five blocks of IGT, dividing both the two groups in subgroups composed of ‘disadvantageous choosers’ and ‘advantageous choosers’. The former PD subgroup chose more frequently the deck with higher immediate profits and low‐frequency but higher losses (which leads to an overall loss), while the latter subgroup selected the deck characterized by immediate but infrequent losses and higher profits (which leads to an overall win); the same was true also for the controls' subgroups. Examining the two PD subgroups, no differences had been detected by taking into account age, clinical history and medication. HC showed a similar behaviour. It was conjectured that performance in IGT might partially be due to the participants' old age (which did not differ among subgroups).

Abbreviations: dlPFC, dorsolateral prefrontal cortex; EEG, electroencephalography; ERPs: event‐related potentials; fMRI, functional magnetic resonance imaging; HC, healthy controls; HD, Huntington's disease; OFC, orbitofrontal cortex; PD, Parkinson's disease; PET, positron emission tomography.

**TABLE 2:** Overview of the performance of PD patients in the IGT as compared with controls (C)

	Stout et al. (2001)	Czernecki et al. (2002)	Thiel et al. (2003)	Perretta et al. (2005)	Mimura et al. (2006)	Pagonabarraga et al. (2007)	Kobayakawa et al. (2008)	Euteneuer et al. (2009)	Kobayakawa et al. (2010)
Blocks of trials measured	4 blocks of 25 trials each	5 blocks of 20 trials each		10 blocks of 10 trials each	2 blocks of 50 trials each	5 blocks of 20 trials each	5 blocks of 20 trials each	5 blocks of 20 trials each	5 blocks of 20 trials each
Money left at the end of the game					PD < C ***p* < .05**		PD < C ***p* < .05**		PD < C ***p* < .05**
Number of advantageous choices in the whole task	PD = C *p* = .70		PD < C *p* > .05	PD < C *p* > .05	PD < C *p* = n.a.	PD < C ***p* < .0001**	PD < C ***p* < .05**	PD < C *p* = .424	PD < C ***p* < .05**
Number of advantageous choices in the second part of the task	PD = C *p* = .44	PD = C *p* = .22		PD < C **Block 7: *p* < .01**	PD < C *p* = .08	PD < C *p* = n.a.	PD < C ***p* < .05**		PD < C ***p* < .05**
Trend (increasing number of advantageous choices along the task) in PD	Yes	Yes		Yes		Opposite	Opposite	Yes	Opposite
Preference for advantageous choices (in the ending of the task) in PD	Yes	Yes	Opposite	Yes	(Slightly) opposite	Opposite	Opposite		Opposite

	Poletti et al. (2010)	Poletti, Frosini, et al. (2011)	Gescheidt et al. (2012)	Gescheidt et al. (2013)	Mapelli et al. (2014)	Buelow et al. (2014)	Xi et al. (2015)	Kobayakawa et al. (2017)	Kjær et al. (2020)
Blocks of trials measured	5 blocks of 20 trials each	5 blocks of 20 trials each	5 blocks of 20 trials each	5 blocks of 20 trials each	5 blocks of 20 trials each	2 blocks: 1–60/61–100	5 blocks of 20 trials each	2 blocks of 50 trials each	5 blocks of 20 trials each
Money left at the end of the game									
Number of advantageous choices in the whole task	PD = C *p* = .318		PD < C Mann–Whitney *U* test ***p* = .048**; Kolmogorov–Smirnov *Z* test *p* = .061	PD < C *p* > .10	PD < C ***p* < .05**	PD < C Selection of deck B: ***p* = .017**	PD < C ***p* = .02**	PD < C ***p* < .01**	PD < C ***p* < .001**
Number of advantageous choices in the second part of the task	PD = C *p* = n.a.	aPD > naPD *p* = n.a.	PD < C Mann–Whitney *U* test ***p* = .033**; Kolmogorov–Smirnov *Z* test ***p* = .047**	PD < C *p* > .05	PD < C ***p* < .01**	PD < C *p* = n.a.		PD < C ***p* < .01**	PD < C ***p* < .05**
Trend (increasing number of advantageous choices along the task) in PD	Yes	Yes	(Slightly) opposite				Yes		(Slightly) yes
Preference for advantageous choices (in the ending of the task) in PD	Yes	Yes	(Slightly) opposite		Yes		Yes	Opposite	

*Note*: In bold: significant results. Blank: not recorded/not reported.

Abbreviations: aPD, alexithymic Parkinson's disease; C, control group; n.a., not available; naPD, nonalexithymic Parkinson's disease; PD, Parkinson's disease patients group.

**TABLE 3:** Overview of the materials and procedures employed in the studies

	Stout et al. (2001)	Czernecki et al. (2002)	Thiel et al. (2003)	Perretta et al. (2005)	Mimura et al. (2006)	Pagonabarraga et al. (2007)	Kobayakawa et al. (2008)	Euteneuer et al. (2009)	Kobayakawa et al. (2010)
Format of the IGT	Physical cards	Computerized	Computerized	Physical cards	Physical cards	Computerized	Computerized	Computerized	Computerized
Features of the cards				Coloured					
Way of responding		Mouse clicking	Button pressing		Turning cards				
Feedback given to the player	Message in the back of the cards	Message on the screen	Told	Black/red labels	Reward/penalty of money	Emoticons and sounds	Reward/penalty of money	Reward/penalty of money	Reward/penalty of money
Form of payment	Play money	Green bar	Play money	Play money	Play money	Play money	Play money	Play money	Play money

	Poletti et al. (2010)	Poletti et al. (2011)	Gescheidt et al. (2012)	Gescheidt et al. (2013)	Buelow et al. (2014)	Mapelli et al. (2014)	Xi et al. (2015)	Kobayakawa et al. (2017)	Kjær et al. (2020)
Format of the IGT	Computerized	Computerized	Computerized	Computerized	Computerized	Computerized		Computerized	Computerized
Features of the cards									
Way of responding									
Feedback given to the player	Reward/penalty of money	Reward/penalty of money	Reward/penalty of money	Reward/penalty of money	Reward/penalty of money	Reward/penalty of money	Reward/penalty of money	Reward/penalty of money	Reward/penalty of money
Form of payment	Play money	Play money	Play money		Play money	Play money	Play money	Play money	Play money

*Note*: Blank: not reported.

**TABLE 4:** Overview of the characteristics of the PD patient groups in the studies where the IGT was employed

	Stout et al. (2001)	Czernecki et al. (2002)	Thiel et al. (2003)	Perretta et al. (2005)	Mimura et al. (2006)	Pagonabarraga et al. (2007)	Kobayakawa et al. (2008)	Euteneuer et al. (2009)	Kobayakawa et al. (2010)
Size of the sample	22	23	5	32	18	35	34	21	14
Mean age (years)	66.0 (57.7–74.3)	57.6 (55.5–59.7)	62.0 (50–74)	72.4–77.7	68.9 (61.9–75.9)	67.2 (63.5–70.3)	69.9 (61–78.8)	67.60 (60.29–74.91)	68.9 (60.9–76.9)
Education (years)	14.2 (11.3–17.1)	11.5 (10.9–12.1)		14.1–14.6		11.9 (11.5–12.4)	13.2 (10,5–15.9)	11.10 (9.21–12.99)	14.8 (10.7–18.9)
Severity of PD (Hoehn and Yahr scale)	2.6 (1.9–3.3)	2.2–3.8	II–III stages	2.1–3.3	II–III stages	2.2 (1.9–2.5)	1.52 (0.77–2.27)	I–III stages	I–III stages
Severity of PD (UPDRS)		12.4–38.7	20.6	11.3–27.2		21.2 (19.6–23.1)		17.7 (8.5–26.9)	
Age of onset of PD (years)	58.3 (50.7–65.9)	42.6 (40.4–44.8)					64.4 (55.2–73.6)		
Duration of PD (years)	7.7 (5.2–13.2)	14.9 (13.7–16.1)	8.0			8.4 (6.4–10.7)	6.4 (3–9.8)	7,14 (1.08–13.2)	5.6 (2.9–8.3)
Dose of l‐Dopa (mg per day)		1115.3 (1047.9–1182.7)		265.6–350.0		645 (405–930)	243 (97–389)	487.69 (170.51–804.87)	476.9 (292.8–661)
Dose of LEDD (mg per day)						226 (198–259)	149 (6–292)		
Total LEDD (mg per day)						870 (603–1189)	391 (178–604)		
MMSE			28.4	Normal	27.8		28.0	29	28.2
Cognitive function	Normal		Normal	Later PD < early PD; C^a^	Normal	Normal	Normal	Normal	Normal
Frontal lobe/executive function	PD > C	PD < C^a^	Impaired	Later PD < early PD; C^a^	PD < C		Normal	PD < C	
Depression	Excluded	Excluded		Excluded but: (Later) PD > C^a^	PD > C^a^	Excluded	PD > C^a^	Excluded but: PD > C^a^	Excluded
Apathy	PD > C^a^	PD > C							
Dysthymia		PD > C							
Alexithymia									

	Poletti et al. (2010)	Poletti, Frosini, et al. (2011)	Gescheidt et al. (2012)	Gescheidt et al. (2013)	Mapelli et al. (2014)	Buelow et al. (2014)	Xi et al. (2015)	Kobayakawa et al. (2017)	Kjær et al. (2020)
Size of the sample	30	24	19	18	15	24	15	20	67
Mean age (years)	64.9 (59.1–70.7)	65.04 (58.81–71.27)	50.32 (41.58–59.06)	52.67 (45.22–60.12)	61.4 (51.8–71)	68.04 (60.18–75.9)	60.73 (48.94–72.52)	70.1 (61.4–78.8)	69.04 (62.74–75.34)
Education (years)	9.3 (5.1–13.5)	8.92 (4.03–12.95)			8.7 (5.1–12.3)	15.63 (12.76–18.5)	8.93 (6.04–11.82)	13.5 (10.8–16.2)	4.29 (1.21–7.37)
Severity of PD (Hoehn and Yahr scale)			1.13–2.27	1.97 (1.42–2.52)		2.36 (1.82–2.9)	1.97 (1.3–2.64)	1.3–2.5	1.94 (1.07–2.81)
Severity of PD (UPDRS)	14.3 (6.2–22.4)		5.9–23.3	18.89 (11.29–26.49)	8.9 (4.9–12.9)	29.73 (20.11–39.35)	15.87 (6.91–24.83)		25.07 (16.84–33.3)
Age of onset of PD (years)	64.9 (59.1–70.7)	65.04 (58.81–71.27)	≤ 45						62.00 (55.37–68.63)
Duration of PD (years)	Newly diagnosed	Newly diagnosed	11.32 (4.9–17.74)	6.33 (3.46–9.2)	4.8 (1.4–8.2)		4.33	6.3 (2.9–9.7)	7.04 (2.92–11.16)
Dose of l‐Dopa (mg per day)			568.4–1949.6			148.61–788.09	122.58–386.78	106.8–421.6	328.77–761.05
Dose of LEDD (mg per day)					334.3–579.7			11.7–226.5	
Total LEDD (mg per day)									328.77–761.05
MMSE	28.8	28.6	29.37	29.7	28.3	28.33	27.6	28.0	27.66
Cognitive functioning	PD = C	Normal	Normal	Normal	PD = C	PD = C	PD = C	PD = C	PD < C^a^
Frontal lobe/Executive function	Normal	Normal	Normal				PD < C	Normal	
Depression	Excluded but: PD > C	Excluded	Excluded	Excluded	Excluded	Excluded but: PD > C	Excluded but: PD > C	PD = C	Excluded but: PD > C^a^
Apathy						PD > C^a^			
Dysthymia									
Alexithymia		aPDA > naPD							

*Note*: Blank: not recorded/not report. Normal: PD patients with no cognitive impairments.

Abbreviations: aPD, alexithymic Parkinson's disease; C, control group; LEDD, Levodopa Equivalent Daily Dose; naPD, nonalexithymic Parkinson's disease; PD, Parkinson's disease patients group.

^a^ The difference was statistically significant.

**TABLE 5a:** Overview of the relationships between performance in the IGT and other measures in PD patients (relationships between the IGT and general characteristics of PD patients)

	Stout et al. (2001)	Czernecki et al. (2002)	Thiel et al. (2003)	Perretta et al. (2005)	Mimura et al. (2006)	Pagonabarraga et al. (2007)	Kobayakawa et al. (2008)	Euteneuer et al. (2009)	Kobayakawa et al. (2010)
Age		− ***ρ* = −.46** ***p* < .05**			n.s.	n.s.	n.s.		n.s.
Education		+ ***ρ* = .45** ***p* < .05**				n.s.	n.s.		n.s.
Severity of PD						n.s.	n.s.	n.s.	n.s.
Duration/onset of PD		n.s.				n.s.	n.s.	n.s	n.s.
Motor impairment		n.s.				n.s.			
Relationships between IGT and PD treatments									
Type of medication	n.s.					n.s.	n.s.		
Dosage						n.s.	n.s.	n.s.	n.s.
Relationships between IGT and other overall measures									
MMSE				n.s.	n.s.	n.s.	n.s.	n.s.	n.s.
MDRS/cognitive impairment	n.s.	n.s.				− ***ρ* = −.56** ***p* = .001**		n.s.	
Executive function	n.s.	− ***ρ* = −.59** ***p* < .01**		n.s.		n.s.		n.s.	n.s.
Depression				+ **Early:** ***r* = .51** ***p* < .05**	n.s.		+ ***ρ* = −.41** ***p* < .05**	n.s.	n.s.
Apathy	n.s.	n.s.							
Alexithymia									
Emotional disorders	n.s.								
BIS‐11 self‐control subscale									

	Poletti et al. (2010)	Poletti, Frosini, et al. (2011)	Gescheidt et al. (2012)	Gescheidt et al. (2013)	Mapelli et al. (2014)	Buelow et al. (2014)	Xi et al. (2015)	Kobayakawa et al. (2017)	Kjær et al. (2020)
Age	n.s.						n.s.	n.s.	
Education	n.s.						n.s.	n.s.	
Severity of PD			n.s.	n.s.	n.s.				
Duration/onset of PD			n.s.	n.s.	n.s.				
Motor impairment					n.s.				
Relationships between IGT and PD treatments									
Type of medication									
Dosage			n.s.	n.s.		n.s.			+ ***r* = n.a.** ***p* < .05**
Relationships between IGT and other overall measures									
MMSE	n.s.					n.s.	n.s.	n.s.	
MDRS/cognitive impairment									
Executive function	n.s.		n.s.				n.s.	n.s.	
Depression	n.s.	n.s.				n.s.	n.s.	n.s.	
Apathy						− ***F* = −.488** ***p* = .005**			
Alexithymia		**+** **2 block:** ***r*=.0559** ***p*=0.013** **−** **4 block:** ***r* = −.488** ***p* = .034** **5 block<:** ***r* = −.666** ***p* = .02**							
Emotional disorders									
BIS‐11 self‐control subscale	− ***ρ* = −.36** ***p* = .05**								

*Note*: Blank: not recorded/not reported. −: negative relation. +: positive relation. In bold: significant results. n.s.: no significant relation.

**TABLE 5b:** Overview of the relationships between performance in the IGT and other measures in PD patients (Relationships between the IGT and other tasks)

	Stout et al. (2001)	Czernecki et al. (2002)	Thiel et al. (2003)	Perretta et al. (2005)	Mimura et al. (2006)	Pagonabarraga et al. (2007)	Kobayakawa et al. (2008)	Euteneuer et al. (2009)	Kobayakawa et al. (2010)
Visual perceptual abilities									n.s.
Digit span						n.s.	n.s.		n.s.
Visual memory							n.s.		n.s.
Auditory/verbal memory		n.s.				− ***ρ* = −.56** ***p* = .001**			
Labyrinths (WAIS)					n.s.				
Stroop				n.s.	n.s.	n.s.			
Tower of London									
Wisconsin Card Sorting/modified version		n.s.		n.s.	n.s.				n.s.
Verbal fluency: Phonemic		n.s.			n.s.	− ***ρ* = −.52** ***p* = .009**			
Verbal fluency: Semantic		n.s.			n.s.	− ***ρ* = −.42** ***p* = .01**			
FAB									
Reading the Mind in the Eyes					+ ***r* = .59** ***p* < .01**			n.s.	

	Poletti et al. (2010)	Poletti, Cavedini, and Bonuccelli (2011)	Gescheidt et al. (2012)	Gescheidt et al. (2013)	Mapelli et al. (2014)	Buelow et al. (2014)	Xi et al. (2015)	Kobayakawa et al. (2017)	Kjær et al. (2020)
Visual perceptual abilities								n.s.	
Digit span							n.s.		
Visual memory									
Auditory/verbal memory							n.s.		
Labyrinths (WAIS)									
Stroop			n.s.				n.s.		
Tower of London			n.s.						
Wisconsin Card Sorting/modified version								n.s.	
Verbal fluency: Phonemic									
Verbal fluency: Semantic									
FAB	n.s.							n.s.	
Reading the Mind in the Eyes							+ ***r* = .58** ***p* = .02**		

*Note*: Blank: not recorded/not reported. −: negative relation. +: positive relation. In bold: significant results. n.s.: no significant relation.

## 3 PERFORMANCE IN THE IGT BY PD PATIENTS

From the description of the studies carried out to investigate the application of the IGT to PD patients (see Table 1), the fundamental question that arises is: As a matter of fact, are PD patients impaired in making decisions in the IGT? Results are controversial. However, whereas in few studies clear differences between PD patients and controls failed to emerge, most reported evidence supporting such differences.

### 3.1 A meta‐analysis

To explore the average true effect in the considered studies regarding the difference in the IGT performance between PD patients and healthy controls, we ran a fixed effect meta‐analysis, using Software R (version 4.1.0), ‘compute.es’ (Del Re, 2010) and ‘metafor’ (Viechtbauer, 2010) packages. We estimated the Cohen's *d* only for those studies in which the means and the standard deviations of the clinical group and the control one in the IGT net score were reported. Otherwise, we used *F* values derived from the difference of the two groups' means. We excluded five studies (Buelow et al., 2014; Kobayakawa et al., 2010; Mapelli et al., 2014; Perretta et al., 2005; Thiel et al., 2003) that did not include any of the main information about the final net scores specified above, as well as Poletti, Frosini, et al.'s (2011) study, which had no healthy control group. For the meta‐analysis, 12 studies were considered (Czernecki et al., 2002.1 reported the ‘first‐on’ condition of the clinical group, whereas Czernecki et al., 2002.2 reported the ‘first‐off’ condition).

The fixed effect analysis showed that the average effect size was −.17 (95% CI: −.34, .01), thus indicating a small effect. We examined the heterogeneity using the *Q* statistic, highlighting a high heterogeneity across the studies at (*I* ^2^) 92.28% (*Q*(*df* = 12= 155.48, *p* < .0001), from which it can be stated that the results across studies showed a considerable variance. After the exclusion of one study (Kobayakawa et al., 2008), which presented an effect size highly different from the others, the heterogeneity remains high (*I* ^2^ = 83.43%; *Q*(*df* = 11) = 66.38, *p* < .0001; ES = −.06, 95% CI: −.23, .12). Heterogeneity can have manifold clinical and methodological causes, that is, the presence of participants' different clinical features or differences in research designs (Del Re, 2015; Higgins et al., 2003), as it has been highlighted in the following sections of the present paper.

**FIGURE 1:**
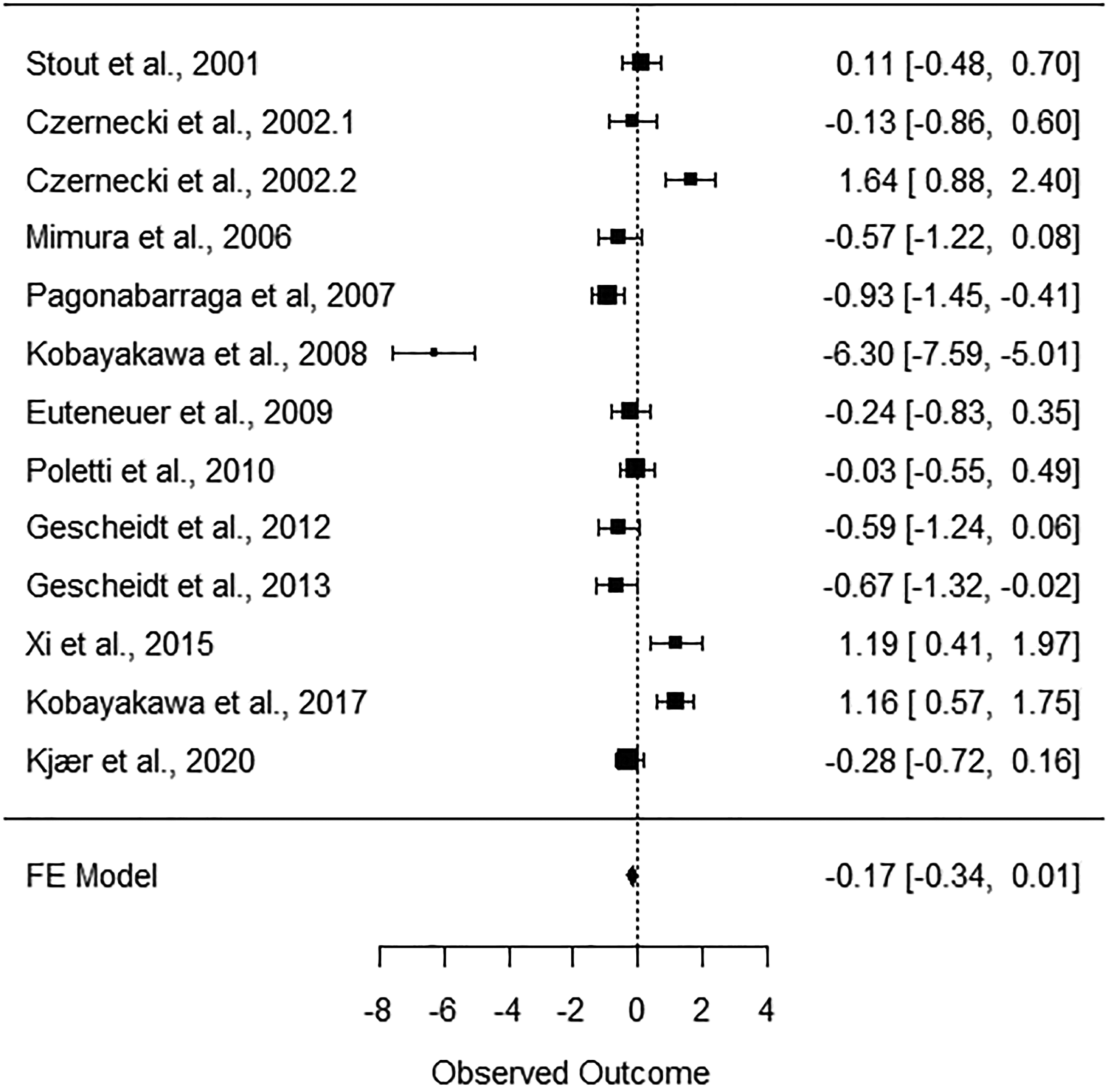
Forest plot of the IGT net score differences between PD group and healthy control group

We reported the forest plot across the studies, with confidence intervals at 95% (Figure 1).

**FIGURE 2:**
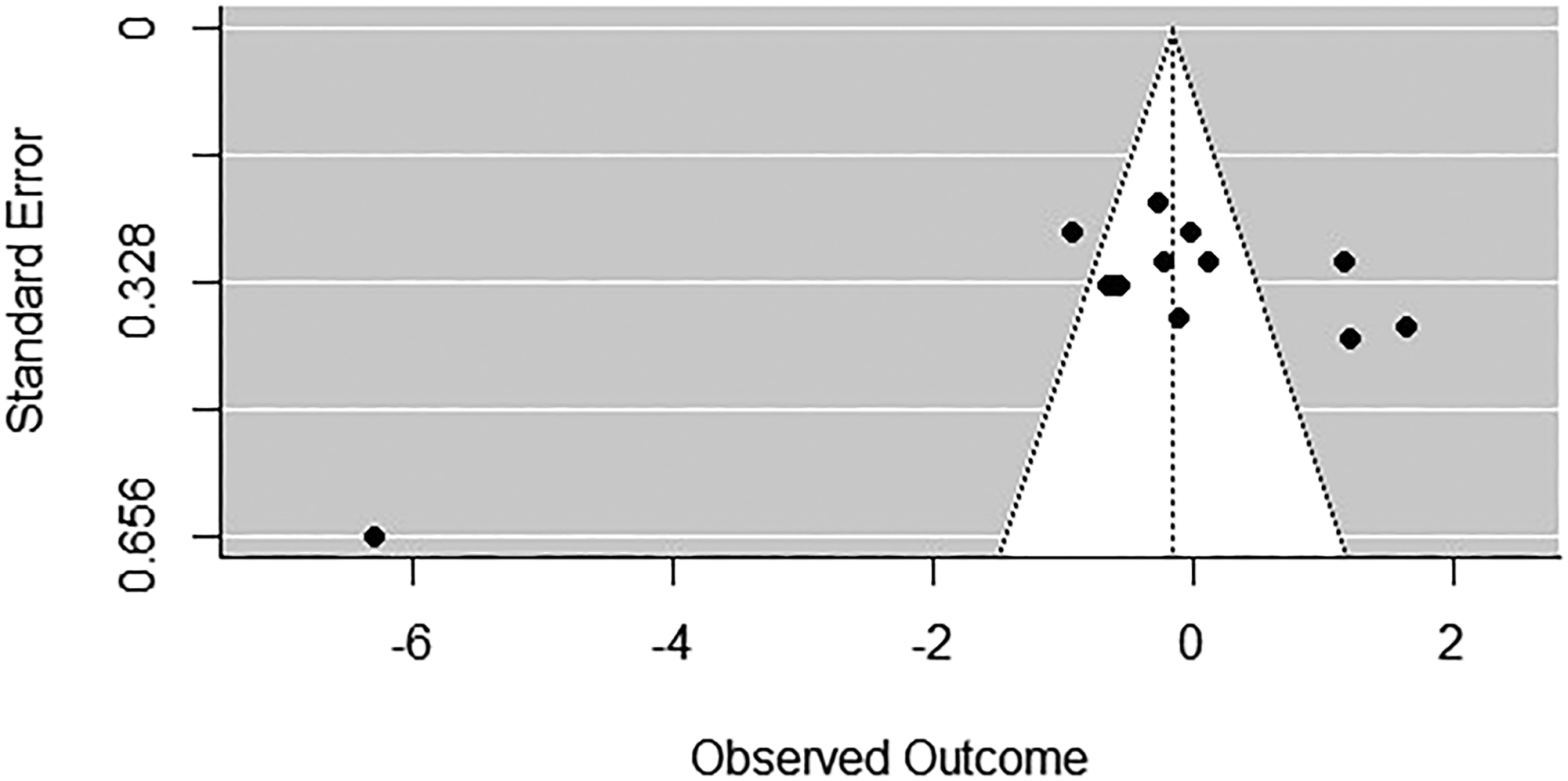
Funnel plot of the effect sizes of the considered studies

Then, we assessed the publication bias using the funnel plot with the trim and fill method, reporting no estimated number of missing studies on the right side (SE = 2.36) (Figure 2).

Thus, we can maintain that, even if a small effect is appreciable, the large heterogeneity of the effects and the restricted number of the studies, which limits the possibility of performing analyses with moderators, suggest that results should be considered with caution. Moreover, not all the studies had used the same parameters to analyse the task or had not reported the required data, so for practical reasons, we considered only the total net score as an indicator of the IGT performance.

Because of the limits that the meta‐analytic approach shows when applied to the set of papers we considered, a complementary approach was followed, consisting in analysing thoroughly the studies to identify possible causes of the inconsistencies that appeared in the findings.

### 3.2 Performance measures

The first aspect that was analysed is the kind of measures that authors computed to assess the performance in the IGT, an aspect that hinders the direct comparisons between the studies because different scoring procedures—as appears from Table 2—were applied.

Only few papers reported the *total amount of money* that players had at the end of the game (Table 2, second row), a measure that expresses the overall efficacy of the decision process. In all cases, PD patients showed a significantly lower amount of money at the end of the game compared with healthy controls (Kobayakawa et al., 2008; Kobayakawa et al., 2010; Mimura et al., 2006). The sum of money left when the game is over depends mostly on the decision of the player to pick up cards from advantageous rather than disadvantageous decks, even if a partial role of chance cannot be discarded (for instance, if the player is lucky, in the last part of the game, he/she may pick up cards with no high penalties from the risky decks, so increasing the total amount of money earned).

Thus, counting the *number of selections* from the two kinds of decks is a more proper measure of the decision behaviour of the participants (Table 2, third and fourth rows). In some studies (Perretta et al., 2005; Thiel et al., 2003) the absolute number of choices of advantageous decks, irrespectively of the number of risky selections, was computed. The same was done also for the number of choices of disadvantageous decks (Kjær et al., 2020). In other studies (Euteneuer et al., 2009; Gescheidt et al., 2012; Gescheidt et al., 2013; Kjær et al., 2020; Kobayakawa et al., 2008; Kobayakawa et al., 2010, 2017; Mapelli et al., 2014; Pagonabarraga et al., 2007; Poletti, Frosini, et al., 2011; Xi et al., 2015), the number of choices of disadvantageous decks was subtracted from the number of advantageous choices in order to have a weighted measure. Globally, PD patients tended to show an overall preference toward disadvantageous decks (even though the effects reached the level of statistical significance in nine out of 14 studies). Also, this measure, however, has the limit to be potentially influenced by chance: In the first phase of the game, the selection of cards from the decks is random, so that a player might select a high number of risky cards without the intention of doing so. The total number of risky choices might be therefore partially influenced by the early, unaware selections. The role of chance can be excluded considering the number of selections *in each block of trials*. Unfortunately, investigators used different criteria to collapse trials in blocks (block sizes ranged from 10 to 60 trials; see Table 2, first row), so preventing us from properly comparing the outcomes of the different studies. Therefore, a valid alternative can be to consider choices in the second phase of the game, namely, when players have acquired enough experience to realize that decks are not equally rewarding/punishing. By counting the number of selections of advantageous choices in the second part of the task (Table 2 fourth row), it appears that the tendency toward this option was lower in PD patients in 11 out of 14 studies, and in six of them, the difference with healthy controls was significant (Gescheidt et al., 2012; Kjær et al., 2020; Kobayakawa et al., 2008; Kobayakawa et al., 2010, 2017; Mapelli et al., 2014), while, among the other studies, it is worth noting that controls chose a higher number of advantageous cards in the middle part of the game (from the 50th to the 70th trials) than PD patients did, with a decrease in the last 30 trials, which approximated their selections to those of patients (Perretta et al., 2005), or a lower performance of patients was recorded only in the second round of the IGT (Czernecki et al., 2002). Instead, Poletti, Frosini, et al. (2011) found that in de novo PD patients, the total mean score of each block tended to increase from the first to the fifth block, but, analysing all the patients, the authors reported that 14 patients recorded a score above 0, whereas 10 patients recorded a score below 0. So, without a healthy control group for comparing the performance, we can only hypothesize a possible tendency to recognize disadvantageous and advantageous decks. In this way, Stout et al. (2001) were the only authors who failed to find a different increase of the rates of selection of safe cards between controls and PD patients along the course of the task.

However, the emerging discrepancy cannot be easily put apart, because we have to highlight other controversial findings concerning the diachronic aspects of players' behaviour across the game. In fact, in three investigations (Czernecki et al., 2002; Poletti et al., 2010; Stout et al., 2001), PD patients showed to have learned to move from the initial disadvantageous choices to an *increased rate of advantageous choices*, a change along the task which is typical in healthy subjects. However, in some studies, the diachronic pattern of performance in the IGT by PD patients did not differ from the healthy controls one, even though the net score by the former group was lower (Euteneuer et al., 2009; Xi et al., 2015). In other studies, although a mild learning effect was appreciable, it was lower than that obtained by healthy controls (Kjær et al., 2020; Mapelli et al., 2014; Perretta et al., 2005). If we focus on the final stage of the IGT, in some studies (Czernecki et al., 2002; Mapelli et al., 2014; Perretta et al., 2005; Poletti et al., 2010; Poletti, Frosini, et al., 2011; Stout et al., 2001; Xi et al., 2015), PD patients finished the game by showing the *preference for advantageous decks*, so proving to be not risk seekers, whereas six studies (Gescheidt et al., 2012; Kobayakawa et al., 2008; Kobayakawa et al., 2010, 2017; Mimura et al., 2006; Pagonabarraga et al., 2007) reported an opposite trend, namely, a progressive increase of risky selections across the game in PD patients (Table 2, sixth row), and two studies described a generalized overselection of risky cards (Buelow et al., 2014; Thiel et al., 2003).

Finally, few studies reported additional measures, as the *number of shifts* from advantageous to disadvantageous cards and vice versa (Gescheidt et al., 2012; Kjær et al., 2020), the *percentage of selection* for each deck in the latter blocks (including Trials 61–100) (Buelow et al., 2014) and the *percentage of used negative feedback* (Euteneuer et al., 2009), consisting in counting every time the participant, after having received a negative feedback, in the following trial did not persist in choosing a disadvantageous option. By considering these measures, it appears that PD patients tend to adopt a more disadvantageous strategy, compared with the healthy controls, both choosing more frequently the decks that led them to a loss (Buelow et al., 2014; Gescheidt et al., 2012; Kjær et al., 2020) and using negative feedback to a lesser extent (Euteneuer et al., 2009) than the healthy control group.

In conclusion, as far as the overall performance in the IGT is concerned, investigations mostly showed that PD patients are less proficient and choose risky decks in the whole game more often than healthy individuals. However, it is unclear if the typical pattern that emerged in healthy subjects—that is, the progressive change from the prevalent choice of disadvantageous decks to that of advantageous decks and the establishment of a risk‐avoidant attitude—fails to be shared by PD patients.

### 3.3 Features of the task

How can we explain the inconsistencies among the studies? The first attempt was to give reasons for such inconsistencies by checking whether methodological issues can explain the differences of the results. Thus, we re‐examined the papers by a thorough analysis of the features of the versions of the IGT applied in the studies, as well as of procedures (as far as they are described in the papers; see Table 3).

A possible variable influencing performance in the IGT was the *format of the game*. However, as appeared by comparing the version consisting of physical cards to be picked up (Mimura et al., 2006; Perretta et al., 2005; Stout et al., 2001) and the computerized, virtual version (where the decks were displayed on a screen, as it appears in most of the studies considered), in the majority of the studies, both versions produced similar patterns of responses (namely, lack of differences between PD patients and controls in the first pair of studies and, at least in part, the presence of differences in the second ones).

Some *perceptual features of the cards* might constitute a facilitation or obstacle to discover the advantageous/disadvantageous nature of the deck. However, when the cover sides of the decks were coloured differently (as specified by Perretta et al., 2005)—a visual aspect that might provide players an implicit hint at identifying the different nature of the corresponding decks and/or might serve as a memory aid—no clear benefit in PD patients was found.

We have no reason to suspect that different *ways of responding*—such as clicking on the mouse (Czernecki et al., 2002) versus pressing a button on a keyboard (Thiel et al., 2003) versus physically turning cards (Mimura et al., 2006)—influenced performance in the IGT, because in all these cases, simple actions to be done with fingers were requested.

It is more likely that the *feedback given after card selection* might affect the identification of the kinds of decks, because such a feedback can reinforce the rewarding versus punishing consequences of the choice. In the majority of the studies (Euteneuer et al., 2009; Gescheidt et al., 2012; Gescheidt et al., 2013; Kobayakawa et al., 2010, 2017; Mapelli et al., 2014; Mimura et al., 2006; Poletti et al., 2010; Poletti, Frosini, et al., 2011; Xi et al., 2015), it is reported that participants won or lost some money after every selection of card, but unfortunately, no additional information has been given. In Stout et al.'s (2001) study, the amount of won or lost related to the chosen card was written behind each of them, and a reward/loss of money occurred immediately after. In Thiel et al.'s (2003) study, participants were told how much money they won and lost, whereas in Czernecki et al.'s (2002) study, such a message appeared onto the computer screen. Gains and losses were written in different colours on the cards employed by Perretta et al. (2005). All these devices seem to play no role in modulating participants' responses. Pagonabarraga et al. (2007) stressed the positive or negative feature of the consequence of the card choice by associating a happy face and a specific sound with the message following a winning choice and a sad face and a different sound with the message following a losing choice. These visual and auditory stimuli reinforce the hedonic valence of the feedback and help the player to remember the consequence of the cards picked up from that deck. However, if we assume that in the papers where this kind of feedback was not described or specified (e.g., Buelow et al., 2014; Kjær et al., 2020; Kobayakawa et al., 2008) it did not occur or it occurred as for the majority of the computerized paradigms, we are induced to figure out that it is not the factor responsible for the differences in PD patients' performance.

Finally, also the *way in which the choices determined changes in the player's financial conditions* was not influential. Similar responses were determined by using play money to remunerate wins and to have money back in case of losses (as it explicitly happened in 14 studies; Euteneuer et al., 2009; Kjær et al., 2020; Kobayakawa et al., 2008; Kobayakawa et al., 2010, 2017; Mapelli et al., 2014; Mimura et al., 2006; Pagonabarraga et al., 2007; Perretta et al., 2005; Poletti et al., 2010; Poletti, Frosini, et al., 2011; Stout et al., 2001; Thiel et al., 2003; Xi et al., 2015) and by showing the increase and the decrease of the player's money after each choice by displaying a green bar onto the computer screen which becomes longer or shorter as a consequence of, respectively, gains and losses (Czernecki et al., 2002).

### 3.4 Participants' characteristics

The second attempt was to check whether the features of the patients (Table 4) could give reason for the inconsistencies in the results. The *size of the sample*—which might explain, if small, the lack of statistical power in testing the differences between patients and controls—seems not to be responsible. It is true that two studies with a large clinical sample (Kobayakawa et al., 2008; Pagonabarraga et al., 2007) brought convincing evidence of a PD patients' impairment in the IGT. However, also in Perretta et al.'s (2005) study, more than 30 patients were recruited, but nevertheless, clear differences failed to emerge.

The inspection of Table 4 suggests that *age* cannot be responsible for the inconsistencies in the results, because clear deficits in PD patients failed to emerge in studies involving patients with both low (Czernecki et al., 2002) and high (Euteneuer et al., 2009) mean ages.

Because of analogous motives, the possible influencing role of education, duration of the disease and dose of medications can be excluded. The mean number of years of *education* was both low (Czernecki et al., 2002; Poletti et al., 2010; Poletti, Frosini, et al., 2011) and high (Stout et al., 2001) in studies failing to show PD patients' impairment in the IGT, as well as in studies reporting such an impairment (Kjær et al., 2020; Kobayakawa et al., 2008; Kobayakawa et al., 2017; Pagonabarraga et al., 2007). The exclusion of age and education is also supported by the lack of correlations between these two variables and performance in the IGT (Table 5a), with the exception of, respectively, the negative and positive correlations found by Czernecki et al. (2002) (it has to be noted that the clinical sample investigated by Czernecki et al. was atypical: It is the only study in which such correlations occurred; Table 5a).

The same was true for *duration of the disease* (low in Stout et al.'s, 2001 study and high in Czernecki et al.'s, 2002 study, both failing to support PD patients' impairment).

Other possible factors influencing the performance in the IGT were the severity of the PD and the age of onset. Indeed, we observe that the impaired responses in the IGT emerged in studies where patients obtained the lowest mean value in the Hoehn and Yahr index of *PD severity* (Gescheidt et al., 2012; Kobayakawa et al., 2008; Kobayakawa et al., 2017). In this way, also when it was higher than 3.0 (both in Czernecki et al.'s, 2002 study about off‐condition patients and in Perretta et al.'s, 2005 studies), the results were not consistent.

If we consider the investigations where the *age of onset* of the illness was recorded, we realize that the expected atypical pattern of responses in the IGT did not emerge or tended to emerge when such age was low, namely, below 45 years old (Czernecki et al., 2002; Gescheidt et al., 2012). When the age of onset was high, the atypical pattern emerged in some cases (Kjær et al., 2020; Kobayakawa et al., 2008) but not in others (Poletti et al., 2010; Poletti, Frosini, et al., 2011). However, regarding this latter comparison, more data are needed, because a large part of the considered studies did not report this piece of information and in the studies conducted by Poletti et al. (2010) and Poletti, Frosini, et al. (2011), the patients showed peculiar conditions (they were de novo patients). In general, neither the severity of the illness nor the age of onset resulted to be correlated to the performance in the IGT (Table 5a).

The *dose of medications* has to be excluded, too. It was low in Perretta et al.'s (2005) study and high in Czernecki et al.'s (2002) study, with both studies showing no significant differences between patients and controls in the diachronic aspects of behaviour held in the IGT. This conclusion is also supported by the findings of the studies explicitly aimed at comparing different pharmacological treatments (Czernecki et al., 2002; Pagonabarraga et al., 2007; Stout et al., 2001), which failed to show differences depending on the pharmacological condition. Only in Kjær et al.'s (2020) study a significant relation between levodopa and dopamine agonist dosage and IGT performance was detected through regression analysis, even if it explained only a very small portion of variance (*R* ^2^ = .04, *F* = 68.28, *p* < .001). So clear conclusions cannot be drawn regarding the possible influence of different pharmacological treatments on the IGT, even if when drug intake had never occurred, patients performed similarly to healthy controls (Poletti et al., 2010), also showing a learning effect (Poletti et al., 2010; Poletti, Frosini, et al., 2011).

*General intellectual efficiency* has to be discarded as a possible explaining factor as well. Mini‐Mental State Examination (MMSE) scores were within the normal range in 10 studies (Table 4, Rows 10 and 11; Buelow et al., 2014; Gescheidt et al., 2012; Gescheidt et al., 2013; Kjær et al., 2020; Kobayakawa et al., 2008; Kobayakawa et al., 2010, 2017; Mapelli et al., 2014; Mimura et al., 2006; Xi et al., 2015) supporting PD patients' impairment in the IGT and in three studies (Euteneuer et al., 2009; Poletti et al., 2010; Poletti, Frosini, et al., 2011) failing to support it. Executive functions (Table 4, Row 12) were less proficient in PD patients recruited in two studies (Mimura et al., 2006; Xi et al., 2015) strongly supporting the impairment and in three studies (Czernecki et al., 2002; Euteneuer et al., 2009; Stout et al., 2001) failing to support it. (The role of cognitive and executive functions in the IGT will be discussed later).

Finally, regarding the emotional states (Table 4, Rows 13–15), most of the studies excluded *depression* or included only patients in the minimal depression range. Where this variable was considered, in a study (Perretta et al., 2005), the tendency to a typical trend in the IGT—namely, the diachronically developing preference for advantageous cards—emerged, whereas in others (Kobayakawa et al., 2008; Mimura et al., 2006), it failed to emerge. The same occurred with non‐depressed patients, who in some cases (Czernecki et al., 2002; Euteneuer et al., 2009; Stout et al., 2001) exhibited the typical behaviour and in other cases (Gescheidt et al., 2012; Kjær et al., 2020; Mapelli et al., 2014; Pagonabarraga et al., 2007; Xi et al., 2015) failed to do so. In this regard, Poletti, Frosini, et al. (2011) investigated differences between mild depressed PD patients and PD patients without this symptom, but no significant differences emerged between the two groups. About the role of *apathy*, which was measured by comparing patients and controls, only few studies considered it (Buelow et al., 2014; Czernecki et al., 2002; Stout et al., 2001) with conflicting results. Other disorders related to emotions, such as *alexithymia* (Table 4, Row 16), were investigated in PD patients only in Poletti, Frosini, et al.'s (2011) study, so no comparison can be done.

### 3.5 Conclusions

The application of the IGT to PD patients revealed in most of the cases that they tend overall to prefer risky choices, even when they might understand that this is not the optimal behaviour to be held. The diachronic development of such a tendency is unclear, with some studies reporting that patients' changes of their choices across trials match what happens in healthy people (namely, the progressive preference toward more advantageous decks and final risk aversion) and other studies showing discrepancies (lack of preference for advantageous choices and lack of risk aversion). Reasons for the controversial findings cannot be found solely in methodological differences due to the materials and/or the procedures. The only factors that might have affected the results seem to concern a feature of the PD treatment: Learning to choose the long‐term remunerating options and avoiding risk—that is, the trends which typically occur in the IGT—failed to emerge when patients had never taken dopamine medications. Unfortunately, only two studies investigated de novo patients (and one of them included no healthy controls), so that we need further studies investigating this peculiar condition in order to exclude a possible role played by other variables (including the fact that de novo patients, being at the initial stage of the disease, can also present a lower cortico‐subcortical impairment, compared with more advanced ones, which can affect the IGT performance, as also Evens et al. (2016) assumed).

## 4 RELATIONSHIPS BETWEEN THE IGT AND GENERAL COGNITIVE FUNCTIONING AND EMOTIONAL STATUS

In several studies, PD patients were asked to perform, besides the IGT, other tasks. Furthermore, the baseline assessment that was carried out in some investigations involved general measures of intellectual efficiency and affective state. The overall consideration of the relationships between IGT and these variables allows us to delve into PD patients' behaviour in the IGT.

### 4.1 Cognitive functioning

As far as *general intellectual functioning* is concerned (Tables 5a and 5b), we have already noted that in some studies, where the preliminary assessment of the levels of mental functioning showed that clinical subjects were less efficient than controls, results about difference in the diachronic pattern of response in the IGT between the two groups were not consistent. In addition, MMSE scores never resulted to be correlated to the IGT and Mattis Dementia Rating Scale (MDRS) or other screening tests for dementia failed to be correlated to the IGT in the majority of cases. The only significant relationship was found by Pagonabarraga et al. (2007), but it merits some caveats (as well as correlations between the IGT and both memory and verbal fluencies; see later), because it emerged only in this study. Thus, the overall picture induces us to maintain that impairments in the IGT exhibited by PD patients are not due to the general intellectual inefficiency.

### 4.2 Emotional disorders

About *depression*, only in two out of 10 studies where this variable was taken into account it was positively correlated to the IGT (Kobayakawa et al., 2008; Perretta et al., 2005). In case of *apathy*, just in one of three studies where this variable was considered, a positive relation with the preference for choosing long‐term disadvantageous decks emerged (Buelow et al., 2014).

### 4.3 The ability to attribute emotions

The final remark concerns the *ability to attribute emotional states*. The positive relation between the IGT and this construct, mainly assessed through the Reading the Mind in the Eyes Test, was strongly supported both by Mimura et al. (2006) and by Xi et al. (2015), whereas it was not supported by Euteneuer et al. (2009). Moreover, regarding *alexithymia*, only one study investigated this variable, in which a positive correlation was found (Poletti, Frosini, et al., 2011).

## 5 TOWARD AN EXPLANATION

Might the patterns of relationships between the IGT and other variables help us in understanding the possible reasons of the impairments in decision processes which were observed in PD patients? As acknowledged by many authors (e.g., Busemeyer & Stout, 2002), the IGT activates a plurality of mechanisms. By summarizing the different attempts made to provide a list of the mental processes involved in the task, we can argue that performing the IGT successfully requires three general kinds of skills:

1. *Emotional skills*: The player should both be sensible to the concrete/emotional value (rewarding or punishing) of the outcomes and try to avoid losses and increase gains.
2. *Cognitive skills*: The player is asked to focus and maintain attention on the decks, to remember the choice of the decks he/she did and the consequent outcomes and to identify the relationships between the kind of decks and the corresponding kind of outcomes (contingency learning).
3. *Executive skills*: The player is requested to suppress the tendency to be attracted by high possible gains (control of impulsivity and recklessness), to plan choice strategies which allow him/her to obtain long‐term benefits and to shift from choices privileging high immediate gains to choices guaranteeing delayed cumulative gains.

The IGT requires a multiplicity of complex skills, essential in decision‐making processes, in order to understand, identify and integrate relevant information toward designated goals and inhibit impulsive replies (Finucane & Gullion, 2010). So a focus on the cognitive abilities underlying them is needed.

### 5.1 Emotional components

As far as emotional skills are concerned, it was argued that failures in the IGT may depend on the insensitivity to reward and punishment and on the absence of care about losses (Dunn et al., 2006; Witt, 2007). Functional magnetic resonance imaging (fMRI) highlighted significant differences between PD patients and healthy participants in functional connectivity of specific structures involved in limbic loop following a punishment but not a reward. In particular, when a punishment occurred, the connectivity between the left anterior cingulate cortex and the right globus pallidus internus was decreased in PD patients, whereas it was increased in healthy controls (Gescheidt et al., 2013). Coherently, analysing event‐related potentials (ERPs), Mapelli et al. (2014) found differences between the clinical group and the control one in processing feedback in the IGT. In detail, in the latter group, there were significant differences in the ERPs' morphology after loss and win outcomes, whereas in the former group, the recorded morphology was the same both after a reward and a punishment. Further evidence is provided by the results about the lack of the typical emotional psychophysiological response, revealed by skin conductance, following the choice of risky decks in PD patients (Euteneuer et al., 2009; Kobayakawa et al., 2008).

From these data, we can conjecture that is not a general indifference about what occurs (a trait which is assessed by the self‐report instruments mentioned above), but an ineffective way to process different feedback and the reduced emotional reaction to events, which have relevant positive or negative consequences for the individual during the task, to be involved. The motivation to maximize the performance in the IGT may be reduced because of the emotional insensitivity to the expected outcomes of one's own choices (Xu et al., 2013).

### 5.2 Cognitive components

Regarding cognitive skills, *attention*, even though never directly tested, seems to play a minor role in the IGT because each trial should stimulate the player's arousal due to the fact that he/she is asked to make a decision. Furthermore, if players ceased to think of the choice to be made because of the drop of attentional resources and interest or boredom and tiredness, selections should become erratic (Yechim et al., 2005), whereas studies reported that not‐random trends emerged.

*Memory* might be involved in the IGT, because if the player focuses only on recent events and forget past events, he/she fails to have in mind a sufficiently broad repertoire of selections, which is the basis to identify the specific features of the decks (Yechim et al., 2005). However, the accurate assessment carried out in some studies by administering memory tests showed that verbal and visual memory skills are not related to the IGT (with the exception of the negative correlation between the IGT and verbal memory found by Pagonabarraga et al., 2007).

A further possibility is that PD patients are impaired in the IGT either because of their lack of capacity to form stable *associations* between the choices they make and the feedback that they receive as a consequence of their choices (Brand et al., 2004) or because of their possible slower learning, due to difficulties in associating each deck with its benefits and risks (Buelow et al., 2014). To prove the latter hypothesis, Buelow et al. (2014) added 100 additional trials to the IGT to test whether patients only need more trials to associate negative and positive outcomes to the corresponding decks. It emerged that whereas in the prior traditional 100 trials, PD patients significantly preferred one of the two disadvantageous decks, in the additional trials, they preferred the other disadvantageous one, so showing that it is not a matter of further opportunities to learn deck–outcome associations. However, when authors split the PD group into apathetic and non‐apathetic patients in their analyses, only the former group obtained significant differences regarding the preference for disadvantageous decks in both the traditional and the additional trials, suggesting that the emotional state may contribute to learning.

Possible impairments in contingency learning were tested both by Czernecki et al. (2002) and Perretta et al. (2005). In both cases, participants had to learn to associate symbols to the corresponding events (rewards or punishments in the former and good or bad weather in the latter case). Czernecki et al. (2002) reported data only about participants' performance after they had acquired the symbol‐feedback association, namely, when such an association was reversed; so we cannot know if PD patients were less efficient than controls in learning stimulus–consequence contingencies. Perretta et al. (2005) instead reported data about all the learning process, by showing that late (but not early) PD patients took more time than controls to learn the contingencies, but reaching approximately the same rates of safe responses as controls in the second phase of the task (namely, after 50 out of 100 trials) (we have to keep in mind that in the IGT the critical phase is the second one, after players have experienced a wide series of deck‐wins/losses contingencies). Moreover, performance in the IGT was not correlated to performance in the contingency learning task. We also have to mention that choices producing long‐term negative wins/losses balance and preference toward risky selections were observed by Brand et al. (2004) in PD patients engaged in the Game of Dice Task, a task where the winning/losing probabilities were explicitly set before the game started, and so no stimulus–consequence contingencies had to be inferred. Thus, in PD patients, contingency learning does not seem to be responsible for decision‐making failures. In conclusion, as Kobayakawa et al. (2008) maintained, the lack of correlations between the IGT and cognitive functions in PD patients supports the notion that their anomalies in decision making cannot be attributed to cognitive deficits.

Brand et al. (2004) argued that PD patients' impairment in the IGT is associated with deficits, besides in emotional feedback processing, also in the *executive function*. In fact, Brand, Recknor, et al. (2007) remarked that in normal participants, only performance in the second part of the IGT resulted to be correlated to executive functions and to the Game of Dice Task. These authors, in line with others (Buelow et al., 2014; Ko et al., 2010), claimed that two processes are involved in the IGT:

1. Making decision under uncertainty (when the probability of success/failure associated to the alternatives to be chosen are unknown: It is the case of the first part of the IGT).
2. Making decisions under risk (when probability is known, either because they are explicitly set—as in the Game of Dice Task—or inferred from previous experience: This happens in the second part of the IGT).

Failures in executive function should explain PD patients' impairments in making decisions under risk. However, we have previously observed that in some studies, failures in the IGT emerged both in PD patients with normal and in patients with lower levels of executive function (Tables 5a and 5b). Furthermore, in the majority of cases, measures of executive function did not result to be correlated to the IGT, except in Pagonabarraga et al.'s (2007) study regarding fluency tasks and in Xi et al.'s (2015) one regarding only the semantic fluency. The spurious ‘frontal score’ recorded by Czernecki et al. (2002) has to be excluded because it cannot be interpreted because such an indicator was a mixed, unclear score resulting from collapsing scores obtained in a variety of different executive function tests and from behavioural observations. In this way, it is worth reporting the conclusions of the study by Poletti et al. (2012), in which de novo patients were split according to their mean total scores into ‘IGT‐fail’ and ‘IGT‐succeed’. There were no significant differences between groups about cognitive functioning, showing that IGT performance cannot be ascribed to classic measures of cognitive functions in which crucial cerebral regions for the IGT—as the OFC—are not involved.

Some authors argued that the way in which PD patients carry out the IGT is affected by a specific aspect of executive control, that is, the lack of ability to *inhibit* an automatized response. Dunn et al. (2006) suggested that PD patients' failures in the IGT may depend on reversal inability, namely, on a deficit in response inhibition, and Witt (2007) maintained that they depend on impaired shifting from initial random responding to choices based on the advantage criterion. However, some studies lead to cast doubts about such conjectures. Czernecki et al. (2002) reported that PD patients were less proficient than controls in the reversal phase of the contingency learning task they used, that is, when participants, after having learned to associate given symbols to the corresponding rewards or punishments, were faced to further trials in which the stimulus–consequence associations were inverted, and so had to inhibit the tendency to respond according to the previously learned associations. Indeed, we have to note that the inhibition of responses based on previously acquired associations was required also in the extinction phase of the task, where PD patients did not differ from controls. Furthermore, PD patients' performance in the reversal learning task was not associated with performance in the IGT. It is true that Cools et al. (2003) reported poorer performance by PD patients (even only in the ‘off’ but not in the ‘on’ state) in the switch task they employed: However, performance in this task and performance in the decisional task they presented to patients (the box game) were not correlated. Finally, neuropsychological tests involving the inhibition of an automatic (Stroop test) or automatized response and the switch to a new criterion to produce the correct answer (Wisconsin Card Sorting Test and some items of the Frontal Assessment Battery) never resulted to be correlated to the IGT in PD patients. It has also to be noted that the IGT does not actually require shifting, because the player has not developed a specific response tendency before he/she realizes that certain decks are advantageous and consequently comes to select them preferably. Therefore, as it is highlighted by other studies (Oyama et al., 2011; Rossi et al., 2010), it seems that the anomalies in the IGT showed by PD patients cannot be ascribed neither to general executive function deficits nor to specific deficits in response inhibition and shifting.

There is, however, a different kind of inhibition that may play a role in the IGT. This kind of inhibition is different from the inhibition considered above because the latter concerns blocking a response—and eventually shifting to another response—which is emotionally neutral and is acquired through a learning process in which that response had been associated to a given stimulus in a conventional way. In this case, the individual has to inhibit the tendency to expect—on the basis of the prior experience—that the previous stimulus will be followed by a stimulus of the same gender (as in the switch task used by Cools et al., 2003) or that a symbol will be followed by a certain information about whether (Perretta et al., 2005) or by a point to be gained (Czernecki et al., 2002). Different is the case of the inhibition of a response which is emotionally connoted and naturally, but not conventionally rooted in the individual, as the tendency to choose risky options might be (Biassoni et al., 2016; Salvatore et al., 2021). In this case, inhibition concerns impulsivity, which has been shown to be high in PD patients when they have to set a bet (Cools et al., 2003). In the IGT, PD patients exhibited their preference for risk (Dunn et al., 2006), choosing options with higher winning but also with higher punishment. Thus, the failure to shift from risky to safe deck selections can be attributed to an impaired control of impulses (Witt, 2007). This is in line with the negative correlation between the IGT score and the Barratt Impulsiveness Scale (self‐control subscale) found by Poletti et al. (2010). Moreover, the positive association between performance in the IGT and depression reported in some studies (Perretta et al., 2005 for early PD patients; Kobayakawa et al., 2008) is consistent with this picture: Depressed PD patients are less prompted to react impulsively to the options and so the need of inhibiting impulsive responses is low.

### 5.3 Conclusion

The behaviour that PD patients hold in the IGT is the result of two types of dysfunction: an impairment in the reward processing, depending on the lacking sensitivity for negative consequences deriving from risky choices, and an impairment in impulse control (two failures that are shared by pathological gambling, in which the abnormalities in decision making which PD patients show in the IGT are emphasized).

## 6 WHAT NEURAL CORRELATES OF DECISION MAKING CAN TELL US ABOUT PD PATIENTS' PERFORMANCE

Can the understanding of the neurobiological processes associated with the execution of the IGT provide us further insights to better comprehend the reasons for the abnormalities in decision making shown by PD patients? Even though Perretta et al. (2005) conjectured that in the IGT a critical role is played by frontal regions, the actual recording of neurobiological data during the IGT was firstly carried out by Thiel et al. (2003) through positron emission tomography (PET) data. These authors reported the activation of both the cognitive loop (dorsolateral prefrontal cortex [dlPFC]–lateral OFC–left caudate nucleus) and the limbic loop (mesial OFC–anterior cingulate gyrus–ventral striatum–nucleus accumbens) in healthy participants. On the contrary, in PD patients, the cognitive loop was intact whereas the limbic loop was impaired. If we consider other pathologies than PD, we realize that abnormal behaviours in the IGT emerge in patients with dysfunction in the ventromedial prefrontal cortex (vmPFC) (Cavedini et al., 2002; Czernecki et al., 2002; Dagher & Robbins, 2009)—a part of the PFC involved in working memory, executive functions and prediction of long‐term consequences (Balconi et al., 2018)—and in the limbic system (Thiel et al., 2003), involved in feedback processing (Brand, Grabenhorst, et al., 2007). Studies focusing on these two lesion sites, namely, vmPFC and the limbic system, showed no anticipatory electrodermal responses during the IGT before choosing disadvantageous decks (Bechara, 2004; Euteneuer et al., 2009), consistently with Kobayakawa et al.'s (2008) and Euteneuer et al.'s (2009) findings. Moreover, some authors (Clark et al., 2008; Ibarretxe‐Bilbao et al., 2009; Li et al., 2010) found that the number of risky choices is linked to the OFC and the limbic loop plays a key role in the performance of the task. More specifically, according to neuroimaging studies, the limbic loop—bounding the mesial OFC to the ventral striatum and the OFC to the vmPFC—plays a key role in ambiguous situations, such as the IGT (Bechara et al., 1994; Hsu et al., 2005; Lawrence et al., 2009). Thus, we are induced to search possible causes of the peculiarities of decision making in PD in the limbic loop.

### 6.1 The role of the limbic loop in the IGT

Brand et al. (2004) observed that the processing of negative feedback (such as realising to have lost a high amount of money in the IGT) is linked to the limbic loop, even though also the temporopolar cortex and the amygdala (which is involved in affectional‐sensory integration)—which are connected to the OFC—might play a role. The limbic loop connects the OFC to the ventral striatum, and striatal dopamine transmission is important for reward processing and learning contingencies in probabilistic tasks (such as the IGT). The lack of dissociations between decision making and executive function suggests, however, according to Brand et al. (2004), that also the cognitive loop (mainly the dlPFC) is involved. In conclusion, deficits in decision making depend on the frontostriatal loop which connects basal ganglia with both limbic and orbitofrontal projection cortices and dlPFC, in line with conclusions of other studies (Ibarretxe‐Bilbao et al., 2009; Li et al., 2010; Salvatore et al., 2021; Xi et al., 2015). These arguments were refined by Brand et al. (2006), who claimed that in the first part of the IGT—when players have to realize that the decks have distinctive rewarding/punishing features—deficits in reversal learning (associated with vmPFC) are relevant; in the second part of the game, once such features have been identified (namely, when it is only a matter of risk, but not of uncertainty) and long‐term strategies have to be applied in a stable situation, executive functions (associated with the dorsolateral sections of the PFC) play a role. In sum, in taking a decision under ambiguity or uncertainty the major role is played by the OFC/ventromedial cortex, whereas deciding under risk involves both the OFC/vmPFC (associated to feedback processing) and the dlPFC (associated to the executive function; Colombo et al., 2015; Oldrati et al., 2016; Oldrati et al., 2018). Afterwards, decision‐making processes under risk result to be more dependent on executive functions than decision making under ambiguity (Brand et al., 2006; Gleichgerrcht et al., 2010). These conjectures are in conflict with what emerged by analysing thoroughly the behaviour of PD patients during the IGT and the relationships between the IGT and other variables. Reversal learning does not seem to be impaired in PD patients, who usually perform the first part of the IGT as well as healthy people. PD patients—as the overall prevalence of choosing risky decks in the IGT and the lack of risk aversion in the IGT showed—are concerned mostly with dealing with risk rather than with ambiguity or uncertainty. However, the defective management of risk exhibited by PD patients does not depend—as studies showed—on failures on executive function.

Therefore, the dlPFC does not seem to be responsible for the PD patients' deficits in the IGT, as it was assumed by Pagonabarraga et al. (2007), who rather argued in favour of the role of the limbic loop only. They maintained that the behaviour of the PD patients in the IGT reveals a disorder in impulse control due to limbic dysfunction: Excessive or pulsatile doses of dopaminergic stimulation (needed to compensate motor changes) overstimulate the limbic regions, acting as a triggering factor for impulse control. In their opinion, impairments in the IGT do not depend on deficits in the dlPFC (because the IGT performance is not related to executive function tasks, which are associated with it).

### 6.2 The effects of dopamine on the limbic loop

It is worth mentioning the dopamine overdose hypothesis. During the early stages of PD, putamen and dorsal caudate nucleus are more damaged compared with ventral striatum, which results less affected. It is assumed that dopaminergic drugs overstimulate the mesocorticolimbic dopaminergic system (Castrioto et al., 2015), overdosing the ventral striatum (Dirnberger & Jahanshahi, 2013; Gotham et al., 1988), bound to the OFC. Consequently, on one hand, in tasks involving putamen and dorsal caudate nucleus—like set‐shifting ones—performance is relatively preserved, relieving from the effect of medications. On the other hand, in tasks depending on ventral striatum—like probabilistic reversal learning and forms of implicit learning (like those implicating rewards) (Pascucci et al., 2017; Vo et al., 2018)—performance is impaired (Gotham et al., 1988; Hiebert et al., 2019). Accordingly, it implies that also the ability to use feedback may be involved (Di Rosa et al., 2015).

The fact that in Pagonabarraga et al.'s (2007) study IGT scores were negatively correlated to verbal fluency can be explained by assuming that verbal fluency is a distinct aspect of executive function, which is associated to inferior frontal gyrus, an area not involved in inhibition. We observed that this ad hoc explanation is not needed if the correlation between the IGT and verbal fluency is conceived as an isolated finding which emerged only in that study.

The role of the limbic loop in impaired performance in the IGT was stressed also by Torta and Castelli (2008). They reminded that PD is associated to a cell loss in the dopaminergic neural population in the mesolimbic and mesocortical networks. Such loss, even though less pronounced, occurs also to the ventral tegmental area projecting to the nucleus accumbens, the amygdala and the prefrontal cortices (including the OFC). Furthermore, the mesolimbic pathways are activated when a reward can be anticipated, but in PD patients, a striatal hypoactivation was observed in this case. In addition, the anticipation of a reward is reduced in PD, as revealed by the amplitude of the stimulus preceding negativity (SPN), a brain potential that occurs in the case of the anticipation of a motivationally relevant event. According to these authors, the neural systems associated to reward anticipation are impaired in PD, as it was mentioned previously. Reward anticipation is conceived by the authors as follows. When the individual has to make a decision, he/she represents to himself/herself the possible outcomes so to be compared with information about internal states and current goals. Such an integration yields an outcome expectancy (namely, the representation of what it is likely to occur as a consequence of the decision), which is related to the OFC. In particular, midbrain dopamine neurons respond to cues that predict reward and to reward prediction errors (e.g., unexpected delivery of reward) (Doya, 2008). Neural activity related to errors in reward prediction has been recorded in the striatum, and it is known that the anterior insula and the lateral OFC respond to variance in predicted reward, as well as that OFC is associated with risk taking and exploration when action outcomes are uncertain. Furthermore, dopamine in the anterior cingulate cortex plays a role when a high reward is expected, so to motivate the subject to choose an action requiring costs. The OFC is also connected with the generation of emotions related to anticipate future events in making advantageous choices (Bechara, 2004). In particular, lateral OFC seems to be connected to punishment evaluation and recognition (Lawrence et al., 2009), whereas medial OFC to reward monitoring, important for choosing between immediate and delayed reward or punishment (Elliott et al., 2000; Kringelbach & Rolls, 2004). To confirm the importance of the OFC in decision process under particular conditions, Kobayakawa et al. (2017) showed an association between IGT scores and decrease of the volume in lateral OFC in both the hemispheres only in PD patients' group, who performed worse than the healthy group did.

To summarize, from literature, it emerges that dopamine alterations can disrupt learning from the positive/negative consequences of the actions (Frank et al., 2007; Salvatore et al., 2021). In particular, the basal ganglia in the substantia nigra, which is the primary source of dopamine in the neostriatum, modulates the connections between various parts of the PFC. More in details, these mechanisms may affect the function of the striato‐thalamo‐frontal pathways, with the possibility to impair the function of the orbitofrontal and cingulated loops and to disrupt the information flow to the ventromedial frontal cortex and to the amygdala.

### 6.3 The possible role of the amygdala

Other authors highlighted that not only OFC but also amygdala can play a role in the tendency toward the preference of disadvantageous choices (Kobayakawa et al., 2008; Peters et al., 2013; Tremblay et al., 2014). In particular, the role of amygdala in the IGT was stressed by Kobayakawa et al. (2008). They reminded that patients with ventromedial prefrontal damages can produce skin responses in the IGT, whereas PD patients failed to do so. Furthermore, amygdala is also involved in emotion reactions. When it is damaged, people can show impairments also in mindreading and social cognition, as proved by Mimura et al. (2006) and by Xi et al. (2015). Coherently, Kawamura and Koyama (2007) concluded that decision making in tasks similar to the IGT and mindreading share the same brain mechanism involving the amygdala. According to their explanation, amygdala deficits produce the lack of triggering of emotional states during the IGT, which in turn determines a reduced sensitivity to risk, which induces PD patients to make risky, disadvantageous choices.

### 6.4 Conclusion

The conclusions most studies seem to agree with are (1) the centrality of the limbic system, the basal ganglia and the OFC in decision making involving rewards and feedback processing; (2) the reduced involvement of the dlPFC in the processes underlying the IGT and (3) the possible role played by dopamine medications in decision making under uncertainty conditions, affecting basal ganglia and structures involved in the limbic loop.

## 7 DISCUSSION

Because from literature an inconsistency about the pattern of performance in the IGT emerged, we read in a critical way the studies, with the aim to figure out possible causes of such variability among results, which also clearly appeared through the meta‐analysis that was reported in the present paper.

Wherever possible, we investigated the features of the IGT and the demographic characteristics of the sample: None of the considered variables seems to have an effective impact on the results. Further, we considered the features of the disease: Data failed to show convincing differences depending on them, in line with Evens et al.'s (2016) review. In addition, we considered the dose of medications taken by the patients. Direct correlations with the IGT performance emerged only in the most recent study (Kjær et al., 2020), showing that, at least in part, dopamine intake can have an indirect effect on the IGT.

### 7.1 The role of cognitive functioning

We took into account the possible role played in the PD patients' performance in the IGT by intellectual functioning and the specific cognitive abilities. We found that a particular case was the ability to attribute mental states. In two out of three studies, the ability to recognize emotions in non‐executive and non‐verbal conditions can be related to the effective reference to emotions to generate appropriate judgments in situations requiring choices. These results seem to support Kawamura and Koyama's (2007) conclusions that mindreading ability and decision‐making tasks—such as the IGT—share a common brain mechanism, involving the OFC and basal ganglia (Bodden et al., 2013; Poletti & Bonuccelli, 2012). When damages occurred, there is a lack of triggering emotional states. This, during the IGT, can determine a reduced sensitivity to risk and the consequent bias for PD patients to make disadvantageous choices, accordingly with the assumption that in PD, there is an impairment in the ability to anticipate the unrewarding consequences of risky decisions.

From studies that investigated the IGT performance and its relation with cognitive abilities in patients with focal lesions located in different brain regions, and specifically in PFC, we can note that patients, when compared with healthy controls, showed a worse performance in the IGT, thus confirming that PFC is a crucial region for decision‐making abilities. On the other hand, mixed results emerged concerning the association between the IGT performance and cognitive abilities, with a particular focus on the executive functions, presumably in part due to the different aetiologies of the patients' lesions. In some studies, no correlation was found between the decision‐making performance and these functions (Zinchenko & Enikolopova, 2017). In other cases, authors (Ouerchefani et al., 2019) investigated the IGT dividing the performance into two parts (according to Brand, Recknor, et al., 2007). Regarding the second part, in vmPFC‐lesioned patients, the performance did not correlate with executive functions, whereas in patients with lesions in dlPFC, whose performance did not significantly differ from that of vmPFC patients, it was linked with shifting and planning abilities. Moreover, the authors highlighted that, whereas dlPFC patients acquired explicit knowledge about which were the decks to be selected as advantageous and which to be avoided as disadvantageous, patients with lesions in vmPFC did not. Thus, even if the performance in IGT resulted similar in both the clinical groups, it seems that difficulties in decision making were mainly due to different impairments in the two groups: Whereas vmPFC patients failed both to understand the long‐term consequences based on previous experiences and to predict long‐term consequences of their choices (which may be read as a reversal learning deficits), dlPFC patients failed to implement a consistent and goal‐oriented behaviour, which can be read as a result of executive difficulties related to the IGT performance. However, it is worth noting that these assumptions are to be meant with caution, because we cannot exclude that, at least in part, deficits can be due not only to the specific lesion site but also to possible disconnections between PFC regions (MacPherson et al., 2009). In addition, analysing healthy samples, most of the studies revealed that the IGT performance is linked to executive functions, especially in their components of inhibition, set shifting and planning abilities, mainly considering the second part of the task (Brand, Recknor, et al., 2007; Gansler et al., 2011; Ouerchefani et al., 2019; Suhr & Hammers, 2010). This demonstrates that, in absence of brain lesions, decision processes investigated through the IGT can be associated to executive functions. Hence, an interesting and complex picture emerges from these results, comparing those found in PD patients (which cannot be entirely overlapped to any of the situations reported above about focal lesions, nor to the aetiopathology, namely, focal vs. neurodegenerative): The IGT can result sensitive to detect difficulties in decision making related to impairments attributable to PFC, in which not only the total score or the number of advantageous choices provides important information about patients' cognitive difficulties but also how he/she implements the decision strategy (e.g., asking patients the features of the decks at the end of the task, with the aim of understanding the reason of their choices).

### 7.2 The role of emotions

The affective components may have a role in decision making in cognitively unimpaired people, in which alexithymia, depression and anxiety modulate the decisional process (see, e.g., Kano et al., 2011; Mueller et al., 2010; Siqueira et al., 2018; Werner et al., 2009). Hence, we can expect that PD patients' behaviour in the IGT can be affected by emotional variables.

Concerning emotional disorders, we considered depression, indicating that it may contrast the impulsive decision tendencies of PD patients. In this way, impulsivity should play a role in the IGT, leading to decisions implying higher wins but also high losses, coherently with Poletti et al.'s (2010) results.

A link between the IGT and the ability to recognize emotions was reported by Poletti, Frosini, et al. (2011) concerning alexithymia in de novo patients. Although only this study considered such variable, it is worth noting that the Authors suggested that this ability deserves to be deepened in future investigations.

A better understanding of the emotional components of the IGT would be of great value also for disentangling the relation between motor and non‐motor symptoms in PD, which can affect information processing (Ehgoetz Martens et al., 2015). On this way, it has been described a link between anxiety, axial disturbances and motor symptoms, depression and cognitive functioning, including decision making (Cubo et al., 2000; Šumec et al., 2017). This can be of considerable importance especially in reference to patients' everyday life and the negative consequences that may occur. Understanding more deeply these relationships can contribute to shed a light not only on decision‐making processing but also on innovative ways to manage symptoms in PD, as Šumec et al. (2017) suggested.

### 7.3 The role of dopaminergic treatment

There may also be a more complex relationship between the accounted variables and the individual characteristics of the participants, more specifically differences in the dopaminergic mechanisms.

Evens et al. (2016) argued that an impairment in PD patients is appreciable in the IGT, but there is no evidence that it is related to dopaminergic treatment effects, according to results from few studies that assessed PD patients during the ‘off’ state. Further research is needed, considering that we cannot exclude that dopamine replacement therapy can lead to neurobiological or molecular long‐term effects in circuits processing reward (for neuroplasticity and plasticity in dopamine circuits, see, e.g., Pignatelli & Bonci, 2015; Volkow & Morales, 2015) which may affect the IGT performance.

Therefore, it might be not exhaustive to study only the short‐term effects of withdrawals nor correlations or regressions of performances of PD patients under treatment to clarify the possible role played by dopamine intake on decision making and feedback processing and possible changes on these cognitive processes. This claim, in combination with results from the studies of Poletti et al. (2010) and Poletti, Frosini, et al. (2011) with de novo patients, can provide an interesting picture, in which it is not only the dosage of drugs to be investigated but also the presence or absence of dopamine intake. From these findings, we propose two suggestions for future research. The first one concerns the need, as argued by Kjær et al. (2018), for longitudinal studies to verify the progression of effects of decisional impairments due to medications. In this vein, it would be interesting to investigate these abilities also before the first intake of dopaminergic drugs. The second suggestion, as argued by Salvatore et al. (2021), regards the possibility that further studies would aim to verify if the IGT might be useful to detect impairments before the occurrence of frank motor symptoms, which are unequivocally linked to a dopaminergic dysfunction. This is due to the characteristic of the IGT to be supported by the mesocorticolimbic system, and therefore, it may be sensitive to a loss of dopaminergic neurons.

### 7.4 The role of metacognitive processes

Other variables not taken into account may affect decision‐making processes assessed by the IGT, such as metacognitive abilities (Balconi et al., 2015; Bechara & Damasio, 2002). Metacognition is essential to understand decision‐making processes (Wokke et al., 2020), above all in complex conditions. Studies using fMRI highlighted the activation of the frontoparietal control network, including regions involved in the decision‐making processes per se (such as regions belonging to the PFC) (Qiu et al., 2018). Metacognition includes the ability to evaluate the consequences of the choices, as well as monitoring and control processes (Wokke et al., 2017; Wokke et al., 2020), and so it allows individuals to change their strategies when feedback differs from the objective. It is argued that subjects can fail to discriminate the adequacy of decision process per se and the desirability of the consequence derived by the choice. Hence, if the result is worse than the expected one (like a loss), the decision is judged as a bad one; otherwise, if the result is positive (like a reward), the decision can be valued as a good one (Colombo et al., 2010), even though it takes a loss in a long‐term perspective, as it could happen in the IGT. So also this aspect may be interesting to deepen in future studies.

## 8 CONCLUSION

In recent decades, both clinicians and researchers showed an increased attention toward cognitive, affective and behavioural impairments in PD, including decision‐making processes.

This paper examined and compared results from the studies involving the IGT, one of the most used tasks to assess decision‐making processes in PD patients. Results highlighted the multiplicity of methodologies used in the studies to investigate the performance to the IGT, which is also reflected in the great heterogeneity stressed by the meta‐analysis. Overall, the meta‐analysis showed a weak effect, where patients tended to prefer disadvantageous decks whereas healthy participants chose mostly the advantageous ones. From the analysis of the studies, we can understand that PD may lead people to prefer risky choices, probably due to an impairment in the ability to anticipate the unrewarding consequences of them and/or to an insensitiveness for punishment, as neurophysiological and electrophysiological findings discussed in the paper suggest.

It seems that performance in the IGT is not associated with demographic features or to the level of cognitive and executive functions of PD patients. Instead, some factors until now little investigated may play a role in the IGT and deserve to be deepen in future research: the ability to recognize emotions, the level of impulsivity and the performance before the initial dopamine drugs intake. These factors can open up crucial research perspectives to delve into the mechanisms underpinning decision‐making competencies in PD patients, with the further aim to prevent possible impairments and to support their decisional processes.

Decision‐making abilities play a key role in patients' quality of life and can affect their long‐term goals, likely including therapeutic compliance (Evens et al., 2016; Salvatore et al., 2021). In this regard, it is worth remembering that the use of dopaminergic drugs, in particular dopamine receptor agonists, can lead patients to develop ICDs (e.g., see Maréchal et al., 2015), with detrimental consequences also in everyday life decision making. Accordingly, it may be of interest to do a screening before the beginning of the therapy, using validated tools as the Questionnaire for Impulsive‐Compulsive Disorders in Parkinson's disease (QUIP) (Weintraub et al., 2009). Such instruments, together with the assessment of decision making using the IGT, could be helpful to prevent negative consequences from possible psychiatric conditions in these patients.

The present work differs from other reviews (e.g., Evens et al., 2016), not only because we considered even the latest studies but also we discussed evidence from different points of view, examining, through a careful analysis, features of the IGT, cognitive and emotional components, neuroimaging data, electrodermal responses and neurobiological processes, with the aim to provide a multidomain overview and to shed a light on factors of different nature that may affect the IGT performance in PD patients. With the present work, we wished to offer an integrated and complementary approach to the issue, capturing and discussing also interesting aspects and mechanisms that emerged from the reported studies, in order to grasp possible crucial aspects, until now little considered, regarding decision making in PD patients.

## CONFLICT OF INTEREST

The authors have no conflict of interest to declare, nor personal, commercial or financial relationships that could be construed as a potential conflict of interest.

## AUTHOR CONTRIBUTIONS

Laura Colautti designed the methodology, provided the study materials, curated the data and reviewed and edited the manuscript. Paola Iannello reviewed and edited the manuscript and supervised the study. Maria Caterina Silveri reviewed and edited the manuscript and supervised the study. Alessandro Antonietti conceptualized the study, designed the methodology, provided the study materials, curated the data, reviewed and edited the manuscript and supervised the study. All the authors have written, read and approved the manuscript.

### PEER REVIEW

The peer review history for this article is available at https://publons.com/publon/10.1111/ejn.15497.

## Data Availability

Not applicable (this article reports no primary data).
